# The unconventional TPX2 family protein TPXL3 regulates α Aurora kinase function in spindle morphogenesis in Arabidopsis

**DOI:** 10.1093/plcell/koaf065

**Published:** 2025-03-26

**Authors:** Xingguang Deng, Takumi Higaki, Hong-Hui Lin, Yuh-Ru Julie Lee, Bo Liu

**Affiliations:** Department of Plant Biology, College of Biological Sciences, University of California, Davis, CA 95616, USA; Ministry of Education Key Laboratory for Bio-Resource and Eco-Environment, College of Life Sciences, State Key Laboratory of Hydraulics and Mountain River Engineering, Sichuan University, Chengdu 610064, China; Faculty of Advanced Science and Technology, Kumamoto University, Kumamoto 860–8555, Japan; International Research Organization for Advanced Science and Technology, Kumamoto University, Kumamoto 860–8555, Japan; Ministry of Education Key Laboratory for Bio-Resource and Eco-Environment, College of Life Sciences, State Key Laboratory of Hydraulics and Mountain River Engineering, Sichuan University, Chengdu 610064, China; Department of Plant Biology, College of Biological Sciences, University of California, Davis, CA 95616, USA; Department of Plant Biology, College of Biological Sciences, University of California, Davis, CA 95616, USA

## Abstract

Spindle assembly in vertebrates requires the Aurora kinase, which is targeted to microtubules and activated by TPX2 (Targeting Protein of XKLP2). In Arabidopsis (*Arabidopsis thaliana*), TPX2-LIKE 3 (TPXL3), but not the highly conserved TPX2, is essential. To test the hypothesis that TPXL3 regulates the function of α Aurora kinase in spindle assembly, we generated transgenic Arabidopsis lines expressing an artificial microRNA targeting *TPXL3* mRNA (amiR-*TPXL3*). The resulting mutants exhibited growth retardation, which was linked to compromised *TPXL3* expression. In the mutant cells, α Aurora was delocalized from spindle microtubules to the cytoplasm, and spindles were assembled without recognizable poles. A functional TPXL3-GFP fusion protein first prominently appeared on the prophase nuclear envelope. Then, TPXL3-GFP localized to spindle microtubules (primarily toward the spindle poles, like γ-tubulin), and finally to the re-forming nuclear envelope during telophase and cytokinesis. However, TPXL3 was absent from phragmoplast microtubules. In addition, we found that the TPXL3 N-terminal Aurora-binding motif, microtubule-binding domain, and importin-binding motif, but not the C-terminal segment, were required for its mitotic function. Expression of truncated TPXL3 variants enhanced the defects in spindle assembly and seedling growth of amiR-*TPXL3* plants. Taken together, our findings uncovered the essential function of TPXL3, but not TPX2, in targeting and activating α Aurora kinase for spindle apparatus assembly in Arabidopsis.

## Teaser

TPXL3 targets α-Aurora kinase to mitotic microtubule arrays and activates its function in bipolar spindle assembly.

## Introduction

The spindle apparatus drives the partitioning of the genetic material during eukaryotic cell division. Spindle microtubules are assembled into a bipolar array and converged toward 2 opposite poles. Developing converged spindle poles guides the segregation of 2 chromatid sets to be incorporated into 2 and only 2 daughter nuclei. Formation of the convergent spindle microtubule array does not require the structurally defined microtubule organizing center of the centrosomes as demonstrated by plant cells (Liu and Lee 2022). The Aurora family kinases serve as master regulators of spindle assembly and a wide spectrum of mitotic and meiotic events on the spindle apparatus by phosphorylating proteins associated with the cytoskeleton and chromosomes (Willems et al. 2018). They are discovered as centrosome-associated kinases of Eg2 in frog cells and Aurora in fly cells, respectively, and their functions are indispensable for mitosis (Glover et al. 1995; Roghi et al. 1998). In fact, these Aurora kinases, often known as Aurora A or AURA in animal cells, not only are concentrated at the centrosomes but also decorate spindle microtubules with biases toward spindle poles, and they represent 1 of the 2 (or 3) classes of Aurora family kinases (Carmena and Earnshaw 2003). The Aurora A counterpart in plants is α Aurora that is detected on spindle microtubules during mitosis and plays an essential role in cell division as its loss causes gametophytic lethality in *Arabidopsis thaliana* (Van Damme et al. 2011).

Aurora A is targeted to spindle microtubules and centrosomes by the microtubule-associated protein TPX2 (targeting protein of XKLP2) (Garrido and Vernos 2016). TPX2 also activates Aurora A by pulling on an activation segment of the kinase and locking it in the active conformation (Bayliss et al. 2003). The vertebrate TPX2 is one of the most important proteins for mitosis because it regulates functions of many spindle assembly factors, e.g. acting in a Ran-GTP-dependent pathway for microtubule nucleation and polymerization, besides targeting and activating Aurora A (Cavazza and Vernos 2015; Heald and Khodjakov 2015; Alfaro-Aco et al. 2017). Through direct interaction, TPX2 also plays a critical role in targeting the Kinesin-5, an essential mitotic motor, to spindle microtubules for the organization of the 2 poles (Ma et al. 2011). TPX2 is conserved in plants as referenced in *A*. *thaliana*, and it is joined by a number of homologous proteins that exhibit different degrees of sequence similarity in regions mostly to the N-terminal Aurora-binding site and the importin-binding TPX2 signature domain (Perrin et al. 2007; Evrard et al. 2009; Tomastikova et al. 2015). Over-expression of TPX2 induces perinuclear and intranuclear microtubule formation possibly in a Ran-dependent manner in *A*. *thaliana* (Petrovska et al. 2013), which seems to suggest a critical function of this highly conserved protein in acentrosomal microtubule nucleation. This assumption was echoed by microinjection of antibodies against animal TPX2 proteins into mitotic cells of *Tradescantia virginiana* (Vos et al. 2008). However, we recently demonstrated that TPX2 is dispensable for normal growth and reproduction as multiple null *tpx2* mutants resembled wild-type control in all examined aspects of growth and development in *A*. *thaliana* (Boruc et al. 2019). This finding challenged the commonly perceived role of canonical TPX2 in mitosis, especially regarding its essential function in Aurora activation and spindle assembly.

Our work also revealed 2 closely related TPX2-like proteins TPXL2 and TPXL3 which both contain segments of TPX2-related peptides but distinctly lack the ∼150-amino acid C-terminal segment found in TPX2 for Kinesin-5 interaction (Boruc et al. 2019). TPXL3, but not TPXL2, was found to be an essential protein. Because TPXL3, like many other TPXL proteins, contains the N-terminal Aurora-binding motif, it can activate the kinase activity of α Aurora in vitro. The expansion of the TPX2 family in plants, as exampled in *A*. *thaliana* (Dvorak Tomastikova et al. 2020), implies potential functional diversification among its members. Because the canonical TPX2 but not TPXL3 is dispensable in *A*. *thaliana* (Boruc et al. 2019), it is unclear whether TPXL3 serves as the primary regulatory protein of the α Aurora kinase in vivo and how its essential function is expressed.

To overcome the obstacle of null mutation-caused lethality and discover the mitotic function of TPXL3, we generated artificial microRNA lines in which TPXL3 expression was repressed. These miR-*TPXL3* lines revealed that TPXL3 plays an essential role in regulating α Aurora activities on spindle microtubules and consequently its function in spindle morphogenesis. Our results showed that morphological changes in mitotic spindles have a great impact on the robustness of plant growth as demonstrated by manipulations of the functions of TPXL3 in transgenic plants. Hence, flowering plants employ a TPX2-independent and TPXL3-dependent mechanism of mitotic regulation.

## Results

### Repression of TPXL3 expression leads to retarded growth

**Figure 1.**
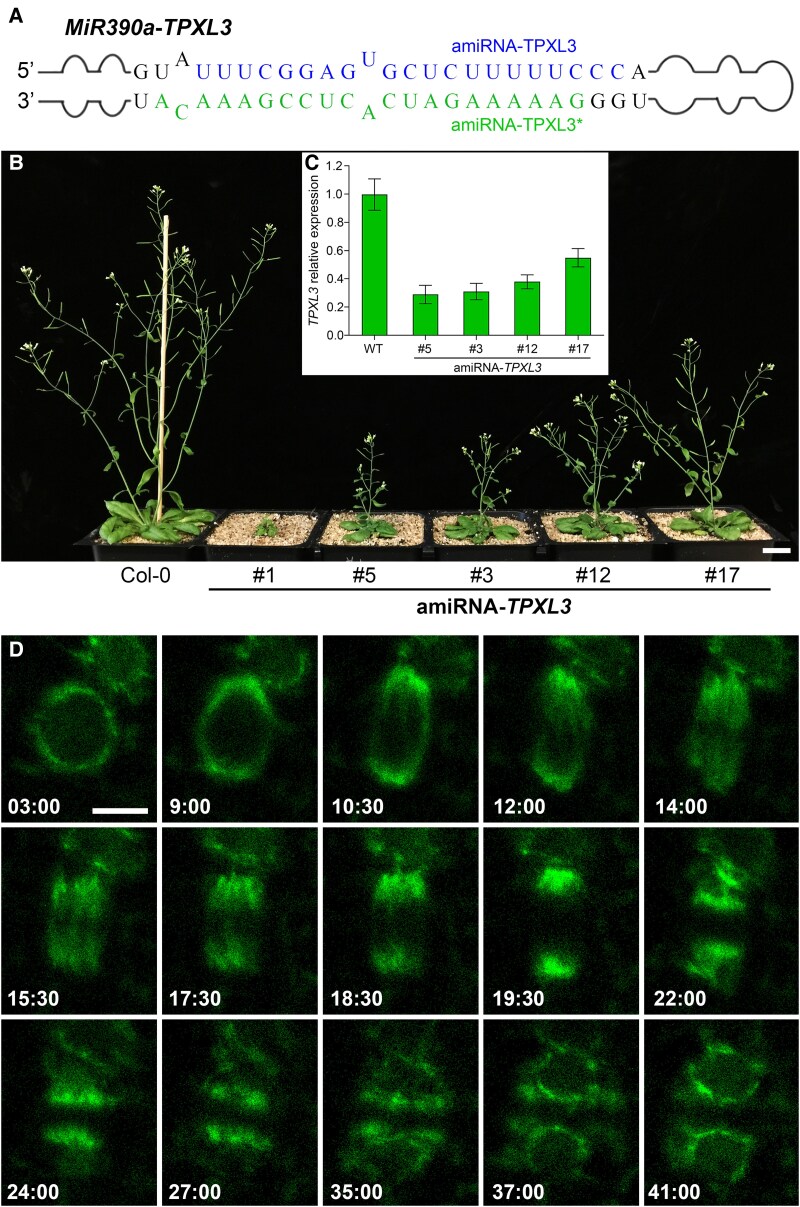
Retarded growth caused by repressed *TPXL3* expression and live-cell imaging of TPXL3-GFP. **A)** A chimeric MiR390a-based construct forms a hairpin structure via the amiRNA-*TPXL3* and amiRNA-TPXL3* sequences. **B)** Growth phenotypes of 9-week-old plants of the wild-type control (Col-0) and 5 independent amiRNA-*TPXL3* transformants. **C)**  *TPXL3* expression was repressed at different degrees in 4 amiRNA-*TPXL3* lines as determined by real-time RT-PCR. Each genotype has a sample size of 9. Bars represent means ± S.D. **D)** Snapshots of TPXL3-GFP dynamics (from Supplementary Video S1) illustrate characteristic localization patterns from prophase to cytokinesis in a dividing cell. Scale bars, 2 cm (**B**) and 5 *µ*m (**D**).

To repress the expression of *TPXL3* in *A*. *thaliana*, an artificial microRNA gene was designed to specifically target this gene but not *TPX2* or other *TPXL* genes (Fig. 1A). The resulting amiR-*TPXL3* transformants produced dwarf plants that exhibited different degrees of growth inhibition when compared to the wild-type control plant (Fig. 1B). To test whether the growth phenotype was correlated to the expression level of the target gene *TPXL3*, real-time RT-PCR experiments were carried out and showed that greater reduction of the mRNA level was associated with more severe growth defects (Fig. 1C). Transformant # 5 (Fig. 1B) was chosen in experiments hereafter. To further prove the linkage of the amiR-*TPXL3* expression with the phenotype, we constructed an amiR-*TPXL3*-resistant version of the *TPXL3* gene (*TPXL3^R^*) by introduction of silent mutations and delivered it into one of the amiR-*TPXL3* mutant (Supplementary Fig. S1A). Expression of the *TPXL3^R^* gene under the control of the *TPXL3* native promoter and in fusion with GFP (green fluorescent protein) fully suppressed the growth phenotype in the mutant to render plants resembling the wild-type control (Supplementary Fig. S1B). Similar TPXL3-dependent growth robustness was also observed in the roots (Supplementary Fig. S1C). Therefore, we concluded that the growth phenotypes exhibited by the amiR-*TPXL3* mutants were caused by the repression of *TPXL3* expression and these mutants could serve as the genetic material for subcellular phenotypic analysis of the consequence of compromised *TPXL3* expression.

To test whether the mutant plants suffered from defects in cell elongation, we used cortical microtubules as a proxy to assess whether they were altered in the mutant cells. Normal transverse microtubules were detected in the live amiR-*TPXL3* cells expressing the visGreen-TUB6 marker, just like those in the wild-type control cells expressing the identical tubulin marker (Supplementary Fig. S2). Downregulation of the microtubule-nucleating augmin function in the amiR-*AUG6* mutant which also showed a dwarf growth phenotype (Liu et al. 2014). Transverse cortical microtubules were detected in the amiR-*TPXL3* cells, similarly as in the control cells, while the amiR-*AUG6* mutant cells had cortical microtubules arranged often in directions along the cell elongation axis (Supplementary Fig. S2A). To compare cortical microtubules quantitatively, we first measured the angle of these cortical microtubules in reference to the orientation perpendicular to cell elongation. The wild-type control and amiR-*TPXL3* cells did not show a significant difference while the amiR-*AUG6* cells clearly had cortical microtubules assuming greater angles (Supplementary Fig. S2B). To test whether there was a difference in the bundling of cortical microtubules, we used Coefficient of Variation (CV) of intensities as the metric as described previously (Higaki 2017). We found that cortical microtubules in all 3 genotypes showed no significant difference (Supplementary Fig. S2C). These results suggested to us that the retarded growth phenotype exhibited by the amiR-*TPXL3* plants was unlikely caused by misbehavior of interphase microtubule activities.

### *TPXL3* exhibits a distinct dynamic localization pattern during mitosis

A previous interaction assay in yeast revealed that TPXL3 barely binds to α Aurora but that the homologous TPXL2 interacts strongly (Boruc et al. 2019). However, it was hypothesized that the TPXL3 protein serves as the primary activator of α Aurora (AUR1 and AUR2) in *A*. *thaliana* in part because of the lethality caused by the loss of this gene. To learn their in vivo activities, we compared the localization patterns of TPXL3 and TPXL2 when fused with GFP and expressed in the corresponding homozygous mutant background. When surveyed by fluorescent microscopy, the 2 proteins exhibited different localization dynamics in dividing root cells (Supplementary Fig. S3A). While TPXL3 showed a pronounced association with mitotic microtubule arrays, TPXL2 did not show obvious localization patterns other than residing in some interphase nuclei. When mCherry-TUB6 was co-expressed with both TPXL3 and TPXL2 in mitotic *Nicotiana benthamiana* cells, respectively, TPXL3 but not TPXL2 was detected on spindle microtubules (Supplementary Fig. S3B). To test whether TPXL2 was associated with mitotic spindles in *A*. *thaliana*, we performed co-immunolocalization experiments with microtubules as the reference and found that there was no concentration of the anti-TPXL2-GFP signal with spindle microtubules when compared to that in the cytosol (Supplementary Fig. S3C). Therefore, this result was in line with the lack of growth phenotype upon the loss of the *TPXL2* gene and supported the essential contribution of *TPXL3* to mitosis (Boruc et al. 2019).

To gain insights into the dynamic localization of TPXL3 during mitosis, we observed the functional TPXL3-GFP fusion protein expressed in the homozygous *tpxl3-1* mutant background (Boruc et al. 2019), by time-lapse live-cell imaging (Supplementary Video S1). The TPXL3-GFP signal became concentrated on the nuclear envelope but not on the preprophase band at prophase and gradually polarized toward 2 poles (min:sec = 3:00 and 9:00, Fig. 1D). Around the time of nuclear envelope breakdown (10:30), the GFP signal was largely concentrated at the poles. Concomitant with the development of the mitotic spindle, the TPXL3 signal spread along spindle microtubules (12:00–15:30, Fig. 1D). Following the anaphase onset (17:30, Fig. 1D), the signal retrieved from the middle zone together with the shortening of kinetochore fiber microtubules and eventually it became highly concentrated at the poles again (19:30, Fig. 1D). When the phragmoplast formed, TPXL3 became enriched in a region at or near the distal ends of the phragmoplast microtubules that faced the daughter nuclei (22:00 to 27:00, Fig. 1D). Such a pattern was later replaced by the returning of the protein to the re-forming nuclear envelope (35:00 to 41:00, Fig. 1D). Therefore, TPXL3 exhibited spindle microtubule-associated distribution pattern during mitosis and a pattern of localization at and beyond phragmoplast distal ends with minimal overlaps with microtubules during cytokinesis, which did not resemble the localization of other MAP proteins.

**Figure 2.**
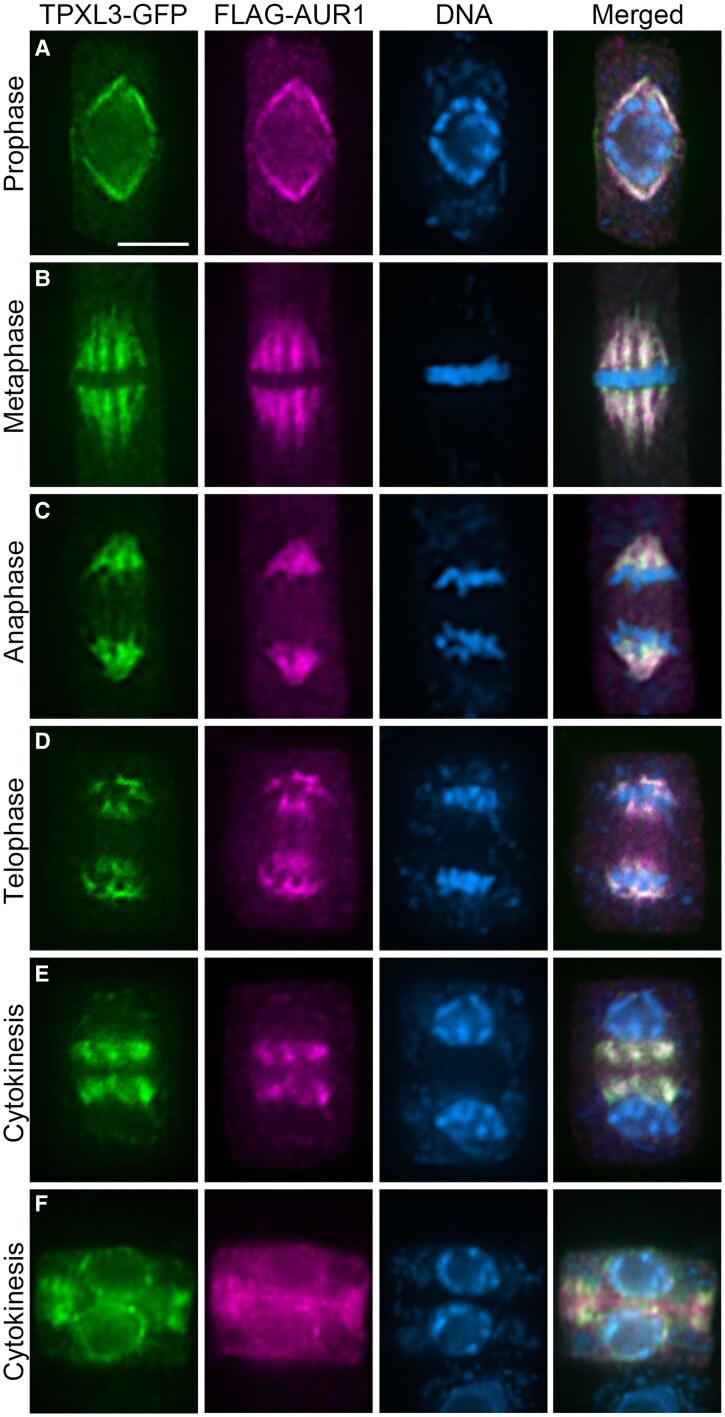
Colocalization of TPXL3 and Aurora1. Immunolocalization images of TPXL3-GFP, FLAG-AUR1, and DNA in mitotic cells at different stages of cell division. The merged images have the 3 colors composited together and the yellow signal represents colocalization of TPXL3 and AUR1. **A)** At late prophase, both TPXL3 and AUR3 are concentrated at spindle poles appeared in the polar caps. **B)** The metaphase cell has both signals that decorate kinetochore fibers. **C)** Both TPXL3 and AUR1 remain at the shortened kinetochore fibers at anaphase and the proteins are barely detectable in the central spindle region between 2 segregating sets of sister chromatids. **D)** Upon the arrival of chromatids at the spindle poles in telophase, remnant signal can be detected at the polar regions while new signal appears in the nuclear region opposite to the poles. **E)** When the daughter nuclei are formed during cytokinesis, both TPXL3 and AUR1 signals become concentrated in areas close to the nuclear surface and facing the opposite nucleus. **F)** When cytokinesis progresses, scarce signals are detected in the phragmoplast region while the nuclear envelope becomes highlighted. Scale bar (applicable to all micrographs), 5 *µ*m.

To discern whether TPXL3 dynamics mirrored that of α Aurora, we then performed colocalization experiments by immunofluorescence in fixed root cells. To do so, the *Aurora 1* (*AUR1*) gene, one of the two α Aurora genes in *A*. *thaliana*, was expressed to produce a fusion protein with the FLAG peptide in the TPXL3-GFP transgenic lines. Like the GFP-AUR1 fusion, this FLAG-AUR1 fusion protein was fully functional as the transgene restored growth of the *aur1 aur2* double mutant to the wild-type level (Supplementary Fig. S4). Throughout the mitotic cell cycle, the FLAG-AUR1 and TPXL3-GFP exhibited identical localization patterns on the prophase spindle as polar caps toward 2 poles (Fig. 2). They were concentrated on kinetochore fibers at metaphase and the association continued on shortening kinetochore fibers at anaphase. At telophase, they were concentrated around the re-forming daughter nuclei (Fig. 2). During cytokinesis, both TPXL3 and AUR1 were concentrated between the 2 daughter nuclei.

To compare the localization pattern of TPXL3 and mitotic microtubule arrays, we performed dual localization of TPXL3 and tubulin (Supplementary Fig. S5A). The TPXL3 signal was particularly pronounced toward spindle poles throughout mitosis. However, it was weakly detected on midzone microtubules during telophase and was not associated with the bulk of microtubules in developing phragmoplasts (Supplementary Fig. S5A). Therefore, the data were consistent with what was detected in vivo.

### Compromised *TPXL3* expression leads to delocalization of α Aurora and γ-tubulin

**Figure 3.**
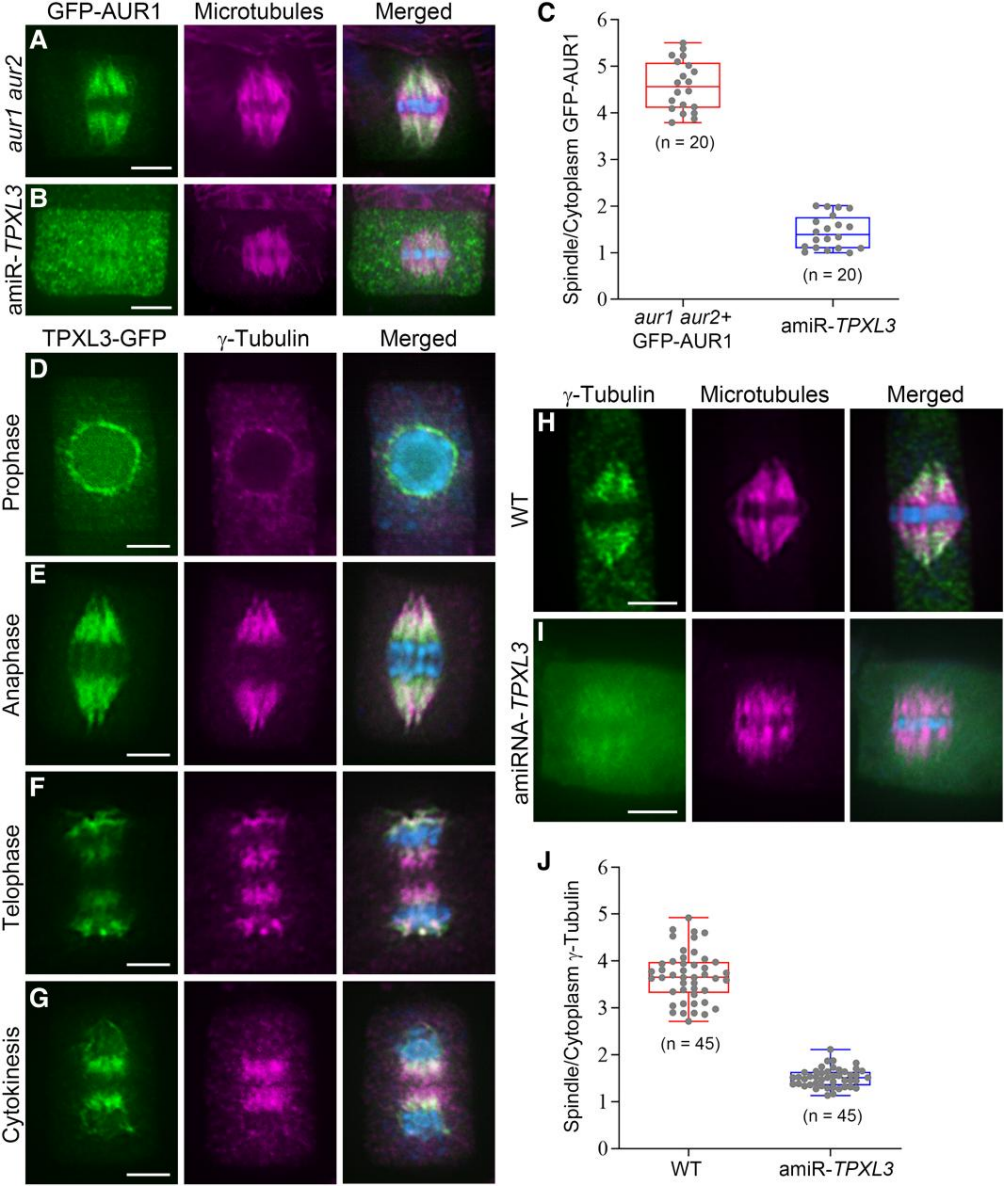
TPXL3 is required for the localization of α Aurora and γ-tubulin in the mitotic apparatus. The merged images have DNA in blue and corresponding green and magenta signals combined. **A)** A functional GFP-AUR1 fusion protein decorates spindle microtubules with biases toward spindle poles, leaving prominent microtubule signal near the metaphase plate in the merged image. **B)** In the amiR-TPXL3 mutant cells at metaphase, the GFP-AUR1 signal becomes mostly diffuse in the cytosol and no longer concentrated along spindle microtubules. **C)** Quantitative assessment shows that spindle-associated GFP-AUR1 signal is greatly compromised and replaced by diffuse cytoplasmic signals as revealed by the ratio of spindle and cytoplasm GFP-AUR1 signals assayed in 20 cells. Data as individual points are presented in box-and-whisker plots, showing the interquartile range (box), the median (horizontal line), and minimum and maximum values (whiskers). **D–G)** Comparative localizations of TPXL3-GFP and γ-tubulin in mitotic cells. At prophase, TPXL3 is heavily associated with the nuclear envelope where γ-tubulin begins to accumulate **(D)**. Both TPXL3 and γ-tubulin are loaded on kinetochore fibers as demonstrated in the anaphase cell **(E)**. At telophase, TPXL3 and γ-tubulin remain at polar regions while weak TPXL3 signal is detected proximal to the chromatid mass, but γ-tubulin is abundantly detected in the central spindle region **(F)**. During cytokinesis when γ-tubulin is heavily associated with the phragmoplast, TPXL3 can only be detected near the re-forming daughter nuclei **(G)**. **H, I)** At metaphase, γ-tubulin localization on spindle microtubules in the control cell is replaced by mostly diffuse signal in the cytoplasm of an amiR-*TPXL3* cell. **J)** Quantitative assessment shows that spindle-associated γ-tubulin signal is compromised and replaced by diffuse cytoplasmic signals as revealed by the ratio of spindle and cytoplasm γ-tubulin signals assayed in 45 cells. Data as individual points are presented in box-and-whisker plots, showing the interquartile range (box), the median (horizontal line), and minimum and maximum values (whiskers). Scale bars, 5 *µ*m.

Because of the colocalization of TPXL3 and AUR1, we asked whether the compromised expression of TPXL3 would affect the localization of AUR1. To do so, we had the GFP-AUR1 fusion protein, which was able to rescue the *aur1 aur2* double mutant (Supplementary Fig. S4), expressed in the amiR-*TPXL3* mutant and compared its localization to that in the control cells. We observed GFP-AUR1 localization in the root tip region of both the control and amiR-*TPXL3* seedling by live-cell confocal microscopy. While the control root tips had cells with concentrated GFP-AUR1 localization toward spindle poles during mitosis and the phragmoplast midline during cytokinesis in all optical sections, the amiR-*TPXL3* roots had cells with diffuse GFP-AUR1 signal (Supplementary Fig. S6). Because AUR1 expressed in a cell-cycle-dependent manner (Van Damme et al. 2011), the cells exhibiting bright GFP signals, representing GFP-AUR1 under the AUR1 promoter, were dividing cells. We also carried out immunofluorescence experiments to compare the localization patterns with greater spatial resolution. In the control cells, GFP-AUR1 prominently decorated spindle microtubules but was absent from the microtubule segments in the vicinity of chromosomes (Fig. 3A). In the amiR-*TPXL3* mutant, however, the GFP-AUR1 signal became largely diffuse in the cytoplasm and its signal on spindle microtubules did not stand out, when compared to the conspicuous association with spindle microtubules in the control cells (Fig. 3B). The contrasted difference was obvious when the spindle-associated signal was compared to and measured against the cytoplasmic one (Fig. 3C). Therefore, we concluded that TPXL3 plays a critical role in targeting AUR1 to spindle microtubules in *A*. *thaliana*.

Conversely, we asked whether compromised α Aurora function could impact TPXL3 localization. We compared the TPXL3-GFP signal in the control cells of the complemented line and in the *aur1 aur2* double mutant background. The localization in the prophase spindle on the nuclear envelope, mitotic spindle, and phragmoplast-devoid region was comparable in both genetic backgrounds (Supplementary Fig. S5B). Therefore, we concluded that TPXL3 likely achieves its localization independently to α Aurora and dictates the localization of α Aurora.

The spindle pole-biased localization of TPXL3 and α Aurora resembled that of microtubule-nucleating factors represented by γ-tubulin in *A*. *thaliana* (Liu et al. 1994). To discern the relationship between α Aurora/TPXL3 and γ-tubulin, we first carried out dual localization experiments by detecting TPXL3-GFP and γ-tubulin with respective antibodies (Fig. 3, D to G). Prior to nuclear envelope breakdown in prophase, TPXL3 prominently accumulated on the nuclear envelope when γ-tubulin was barely detectable (Fig. 3D). After nuclear envelope breakdown, the TPXL3 signal largely overlapped with γ-tubulin on kinetochore fibers of the mitotic spindle (Fig. 3E). Striking differences were discovered in the spindle midzone and developing phragmoplasts where γ-tubulin prominently decorated microtubule minus ends (Fig. 3, F and G). TPXL3, however, was barely detected and later accumulated at the distal ends of the 2 groups of the γ-tubulin signal that faced the re-forming daughter nuclei (Fig. 3, F and G). This finding was surprising because γ-tubulin is highly enriched at the minus end of phragmoplast microtubules. Therefore, perhaps TPXL3 is associated with subcellular structures that are located at the distal ends of the phragmoplast or flank the phragmoplast microtubules.

Because TPXL3 appeared earlier than γ-tubulin on mitotic arrays, we then examined γ-tubulin localization in the amiR-*TPXL3* mutant cells. Compared to the conspicuous localization of γ-tubulin on prophase spindle poles and developing spindles in the control cells, such polarized pattern was largely lost and replaced by weak signals clouding on microtubules (Fig. 3, H and I). The γ-tubulin signal that associated with spindle microtubules was seriously reduced while the diffuse signal in the cytoplasm became more noticeable (Fig. 3J). Therefore, the results support the notion that TPXL3 plays a critical role in regulating the localization of α Aurora and perhaps consequently γ-tubulin complex on the spindle microtubule arrays.

### TPXL3 directly binds to microtubules in vivo and is phosphorylated by α Aurora

**Figure 4.**
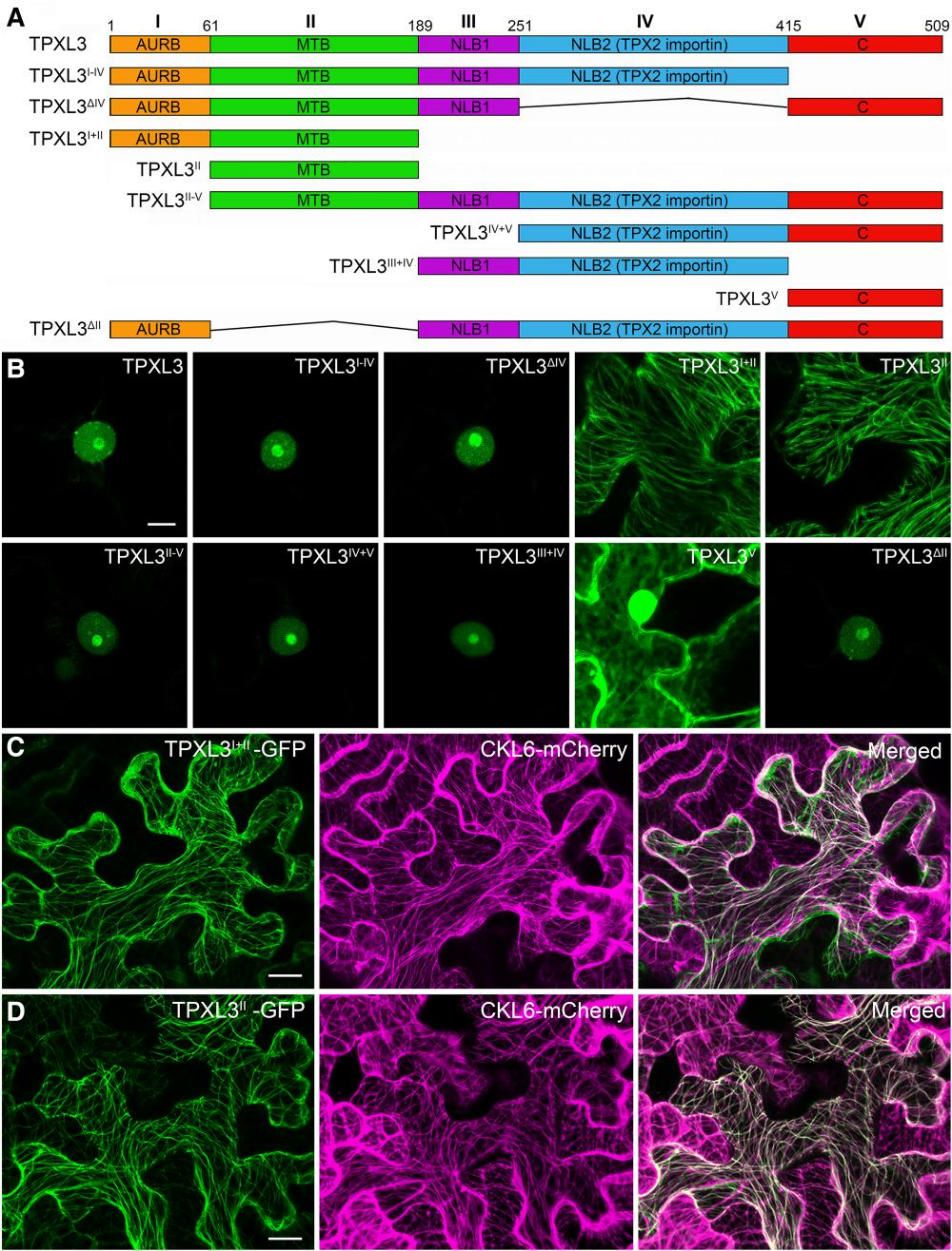
Functional domains of TPXL3. **A)** TPXL3 is divided into 5 domains (I to V). Full-length and truncated versions of TPXL3 are used in later assays. **B)** Transient expression of TPXL3 and its derivatives in interphase cells. The nuclear localization of the protein is retained when either domain III or IV is included. The removal of domains III, IV, and V renders the truncated protein on cortical microtubules, and domain II is sufficient for the association. Domain IV overrides domain II to drive the truncations to the nucleus. The C-terminal domain V assumes a generic nucleocytoplasmic localization pattern. **C, D)** Both the TPXL3^I + II^-GFP and TPXL3^II^-GFP fusion proteins decorate the cortical microtubule network marked by the CKL6–mCherry fusion protein. Scale bars (applicable to all micrographs included), 10 *µ*m.

Because the TPXL3 polypeptide exhibits substantial sequence divergence from the canonical TPX2 protein (Boruc et al. 2019), we dissected the structure-function relationship by transiently expressing truncated versions in *N*. *benthamiana* cells (Fig. 4). To do so, we divided TPXL3 into the following 5 segments (I to V): the N-terminal Aurora-binding motif (I), first previously unannotated domain (II), domain III with the first predicted nuclear localization signal, and domain IV with the second nuclear localization signal and importin-binding site, and the C-terminal previously unannotated domain (V) (Fig. 4A). Interestingly, proteins of the dicot but not monocot origins share noticeable sequence similarity in domains II and V. When the full-length TPXL3 was expressed in fusion with GFP under the control of the constitutive viral 35S promoter, the fusion protein was nuclear with stronger signals in the nucleolus (Fig. 4B). The deletion of either domain V or IV did not alter such a localization pattern (Fig. 4B). The removal of domains III–V, however, resulted in the fusion protein decorating cortical microtubule-like network, and domain II was sufficient for this localization pattern (Fig. 4B). To ascertain whether the cytoskeletal or nuclear localization would dominate when both features were included, domains II–V were expressed and exhibited nuclear localization like others seen above (Fig. 4B). In fact, domains IV plus V, III plus IV, or I plus III to V also rendered similar localization patterns (Fig. 4B). Finally, we tested whether domain V had a localization determinant and found its GFP fusion was uniformly diffuse in the cytoplasm and nucleus (Fig. 4B). To ascertain whether domain II interacted with cortical microtubules, we expressed the truncated protein together with a microtubule marker of CKL6-mCherry (Ben-Nissan et al. 2008) and detected completely overlapping patterns either with domains I and II or with domain II alone (Fig. 4, C and D). Therefore, we concluded that domain II constitutes a microtubule-binding site in TPXL3 while domains III and IV have nuclear localization activities. The essential function of TPXL3 perhaps is brought about by these domains that make respective contributions.

**Figure 5.**
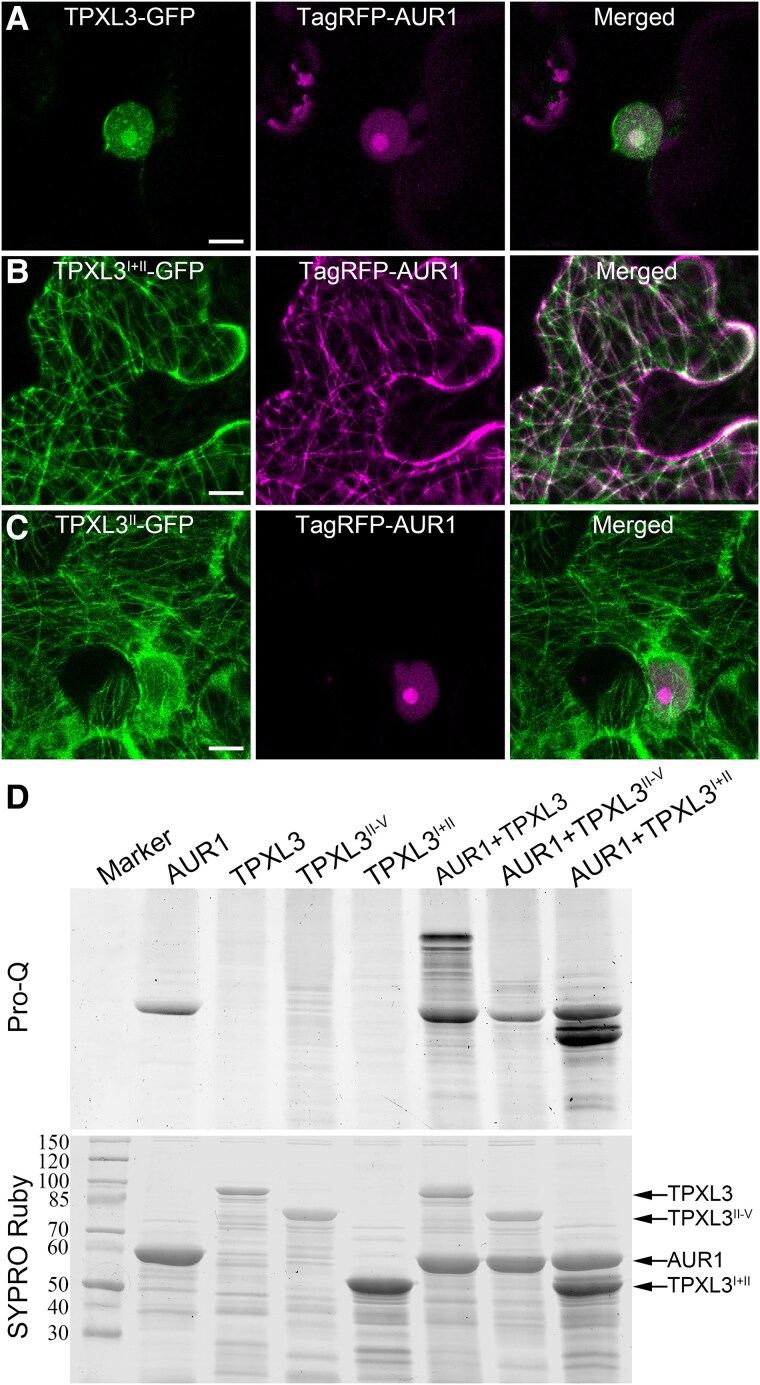
TPXL3 governs α Aurora localization and activates its kinase activity. Colocalization can be visualized in white color in the merged images. **A)** Full-length TPXL3 and AUR1 exhibit nuclear localization upon transient expression in *N*. *benthamiana* cells. **B)** Co-expression of TPXL3^I + II^-GFP and TagRFP-AUR1 relocalize AUR1 to cortical microtubules. **C)** TagRFP-AUR1 remains in the nucleus when it is co-expressed with TPXL3^II^-GFP which decorates cortical MTs. **D)** AUR1 is associated with and activated by TPXL3 as assayed by the Pro-Q phosphoprotein assay. The same gel is stained by SYPRO Ruby to reveal total proteins. Weak autophosphorylation activity of AUR1 is enhanced upon binding to TPXL3 which becomes phosphorylated. Such an effect is dependent on domain I which renders AUR1-binding. The TPXL3^I + II^ truncation is sufficient for both the binding and activation of AUR1. Scale bars, 10 *µ*m.

Both α Aurora and TPXL3 exhibited nuclear localization in interphase cells (Boruc et al. 2019). When co-expressed, the full-length TPXL3 fused with GFP had exclusive nuclear localization with an emphasis in the nucleolus, as the TagRFP-AUR1 fusion protein (Fig. 5A). To test whether α Aurora localization was dependent on TPXL3, we had TagRFP-AUR1 co-expressed with truncated TPXL3 containing only domains I and II corresponding to the Aurora-binding motif and microtubule-binding domain, respectively. Consequently, TagRFP-AUR1 decorated cortical microtubules and completely overlapped with TPXL3^I + II^-GFP (Fig. 5B). Such microtubule localization was completely dependent on the domain I as the loss of this AUR-binding site abolished the microtubule-association (Fig. 5C).

The AUR1–TPXL3 interaction was further tested in vitro using fusion proteins expressed in and purified from bacterial hosts. When compared to the interaction between the full-length GST-TPXL3 and 6xHis-AUR1, it was lost when the GST-TPXL3^II−V^ fusion protein was used (Supplementary Fig. S7). The deletion of domains III to V did not affect the interaction (Supplementary Fig. S7).

We then tested how specific domain(s) of TPXL3 contributed to the kinase activity of α Aurora. AUR1 exhibited some autophosphorylation activities, while full-length TPXL3, TPXL3^II−V^, or TPXL3^I + II^ alone did not (Fig. 5D). The addition of full-length TPXL3-enhanced AUR1 phosphorylation and had TPXL3 also phosphorylated, and such an activity was completely dependent on the Aurora-binding domain at the N-terminus (Fig. 5D). Furthermore, the results also showed that the phosphorylation sites were largely included in the Aurora- and microtubule-binding sites within the first 2 domains (Fig. 5D).

### TPXL3 regulates spindle morphogenesis

**Figure 6.**
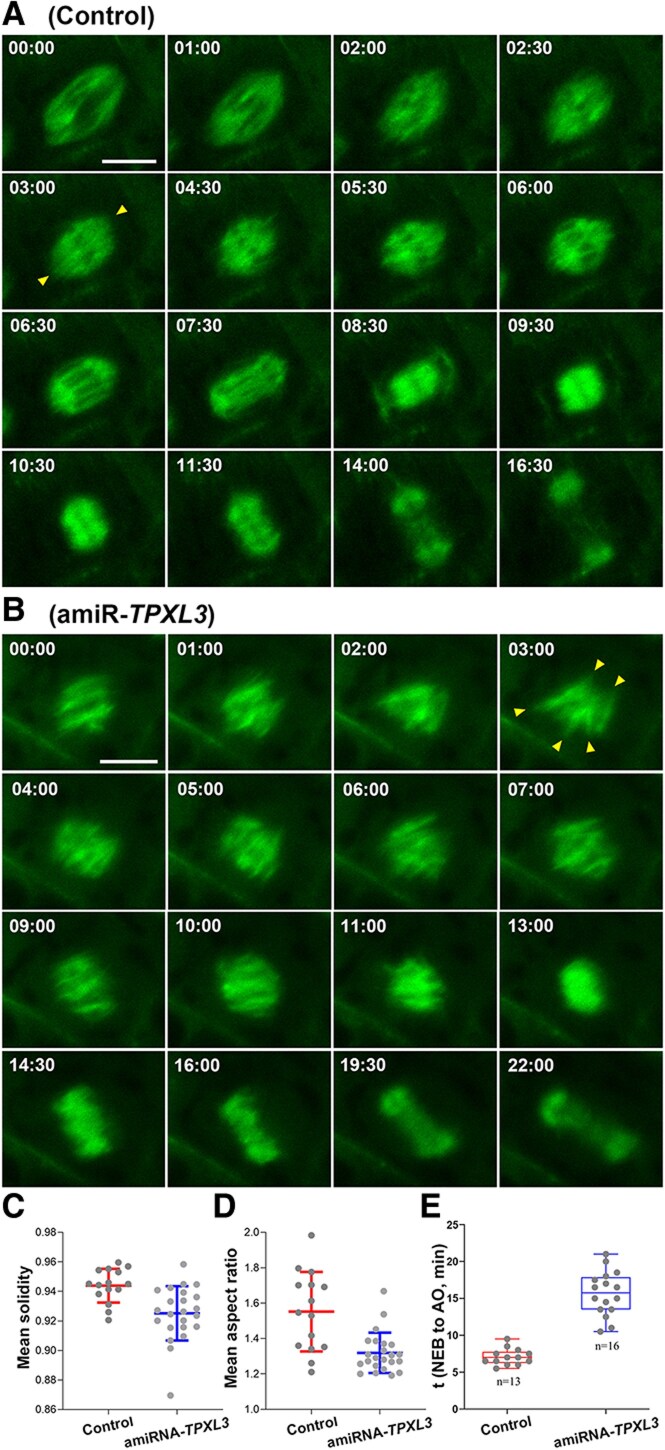
Mitotic microtubule reorganization in the control and amiRNA-*TPXL3* mutant cells. Snapshots are taken from Supplementary movies S2 and S3. **A)** When the control cell enters mitosis, microtubules are organized into a fusiform pattern at late prophase (00:00). Such an array is later replaced by metaphase spindle after the cell establishes all kinetochore fibers that are converged toward spindle poles (arrowheads) (01:00 to 03:00). Spindle microtubules continuously converge toward poles throughout mitosis (4:30 to 7:30). Upon the completion of mitosis, microtubules in the spindle midzone are assembled and arranged in a mirrored configuration with a fluorescently dark line in the middle as a phragmoplast microtubule array (8:30 to 9:30). These phragmoplast microtubules are shortened at distal ends while the array expands toward the cell periphery concomitantly with the disappearance of microtubules toward the center (10:00 to 16:30). **B)** In the amiRNA-*TPXL3* mutant cell, microtubules appear in discrete bundles at late prophase and prometaphase (00:00). In the rest of prometaphase, these microtubules do not form a converged array and point at various directions [arrowheads (1:00 to 3:00)]. Kinetochore fibers are established prior to their shortening during anaphase (4:00 to 7:00). Microtubules in the spindle midzone coalesce following mitosis and are developed into a phragmoplast array that expands toward the cell periphery, similarly as in the control cell (9:00 to 22:00). Data as individual points are presented in box-and-whisker plots, showing the interquartile range (box), the median (horizontal line), and minimum and maximum values (whiskers). **C)** The mean solidity of amiRNA-TPXL3 spindles is significantly reduced when compared to the wild-type control based on 23 and 15 movies with standard deviations are 0.925 ± 0.013 and 0.944 ± 0.082, respectively. Data as individual points are presented in box-and-whisker plots, showing the interquartile range (box), the median (horizontal line), and minimum and maximum values (whiskers). **D)** The mean aspect ratio of amiRNA-*TPXL3* spindles is significantly reduced when compared to the control based on 23 and 15 movies with standard deviations are 1.552 ± 0.186 and 1.319 ± 0.008, respectively. **E)** Comparison of the times from nuclear envelope breakdown (NEB) to anaphase onset (AO). The average times from NEB to AO with standard deviations are 7.08 ± 1.12 and 15.66 ± 3.03 min for the wild-type control and amiR-*TPXL3* cells, respectively. The difference is obvious because the error bars of the control and amiR-*TPXL3* cells do not overlap. Scale bar, 5 *µ*m.

To link TPXL3 function to microtubule remodeling during mitosis, we delivered a GFP-TUB6 marker of microtubules into the amiR-TPXL3 mutant to monitor mitotic arrays and compared them to those in control cells by live-cell imaging (Supplementary Video S2 and S3). Microtubules are assembled into a bipolar prophase spindle around the timing of nuclear envelope breakdown in the control cell expressing TPXL3 (min:sec = 00:00 to 01:00, Fig. 6A). This array was followed by the spindle arrays of prominent kinetochore fibers converged toward obvious poles (02:00 to 06:00, Fig. 6A). Following anaphase onset, converged kinetochore fibers shortened and microtubules in the spindle midzone emerged and coalesced before the appearance of 2 mirrored microtubule sets joined by the fluorescently dark midline (06:00 to 08:30, Fig. 6A). Such a dynamic pattern was largely altered in the amiR-TPXL3 mutant cell although microtubules continued to undergo rapid reorganization (Fig. 6B). Unlike the fusiform appearance of spindle microtubule in the control cells, mutant cells assembled discrete microtubule bundles running in parallel to each other upon nuclear envelope breakdown (00:00, Fig. 6B). These microtubule bundles were not integrated into a spindle with converged spindle poles when bundles freely splayed outwards (01:00 to 03:00, Fig. 6B). Although a bipolar spindle array was formed later with obvious kinetochore fibers terminating at the metaphase plate, it lacked converging poles (04:00 to 07:00, Fig. 6B). Anaphase onset was delayed, and sometimes, anaphase spindle elongation was minimized, and microtubules in the spindle midzone were developed into robust bundles that later coalesced into the phragmoplast array (09:00 to 13:00, Fig. 6B).

To quantitatively evaluate the convergence of spindle microtubules, we measured spindle solidity, which is defined as the ratio of the spindle area to the convex hull area (Fig. 6C, Supplementary Fig. S8, A and B). Converged and splayed microtubules result in high and low ratios, respectively. Repression of *TPXL3* expression significantly decreased solidity, leading to the splayed organization of spindle microtubules (Fig. 6C). To quantitatively evaluate spindle elongation, we also measured spindle aspect ratio, which is a morphometric parameter that is defined as the ratio of the pole-to-pole length to the spindle width in the equatorial plane (Fig. 6D, Supplementary Fig. S8, A and C). Knockdown of *TPXL3* significantly decreased the aspect ratio, leading to decreased spindle elongation (Fig. 6D).

We also compared the times from nuclear envelope breakdown to anaphase onset in the control and amiR-*TPXL3* cells. While the control cells spent shorter than 10 min, the amiR-*TPXL3* cells spent over 15 min (Fig. 6E). Therefore, we concluded that downregulation of TPXL3 expression seriously slowed down mitotic progression.

Although the amiRNA-*TPXL3* mutant cell formed distorted spindle microtubule arrays, it produced a bipolar phragmoplast array that underwent robust expansion toward the cell cortex during cytokinesis, similar to what was observed in the control cell (09:30 to 16:30 in the control and 13:00 to 22:00 in amiR-TPXL3, Fig. 6, A and B). Therefore, we concluded that downregulation of TPXL3 expression had greater impacts on spindle microtubule remodeling than on the phragmoplast array.

We also tested how compromised TPXL3 function could have affected mitotic progression upon challenges with a low dose (100 nm) oryzalin. The mitotic cells of the control plant were able to quickly recover from the oryzalin shock to establish bipolar spindles and entered anaphase within 10 min (Supplementary Fig. S9A). In the amiR-*TPX3* mutant cells, however, microtubule bundles formed after nuclear envelope breakdown appeared in a random organization pattern instead of being engaged in a bipolar array (Supplementary Fig. S9B). Such a microtubule organization defect persisted in the mutant cell for over 16 min after nuclear envelope breakdown. When the amiR-*TPXL3* seedlings were grown on a medium containing 100 nm oryzalin, they showed very little if any root growth when compared to the control, and the phenotype was completely suppressed upon expression of the amiR-*TPX3* resistant form of the protein (Supplementary Fig. S1C). Therefore, it was concluded that the amiR-*TPXL3* mutant had its mitotic cells hypersensitive to such a low concentration of oryzalin that likely attributed to the retarded root and seedling growth.

**Figure 7.**
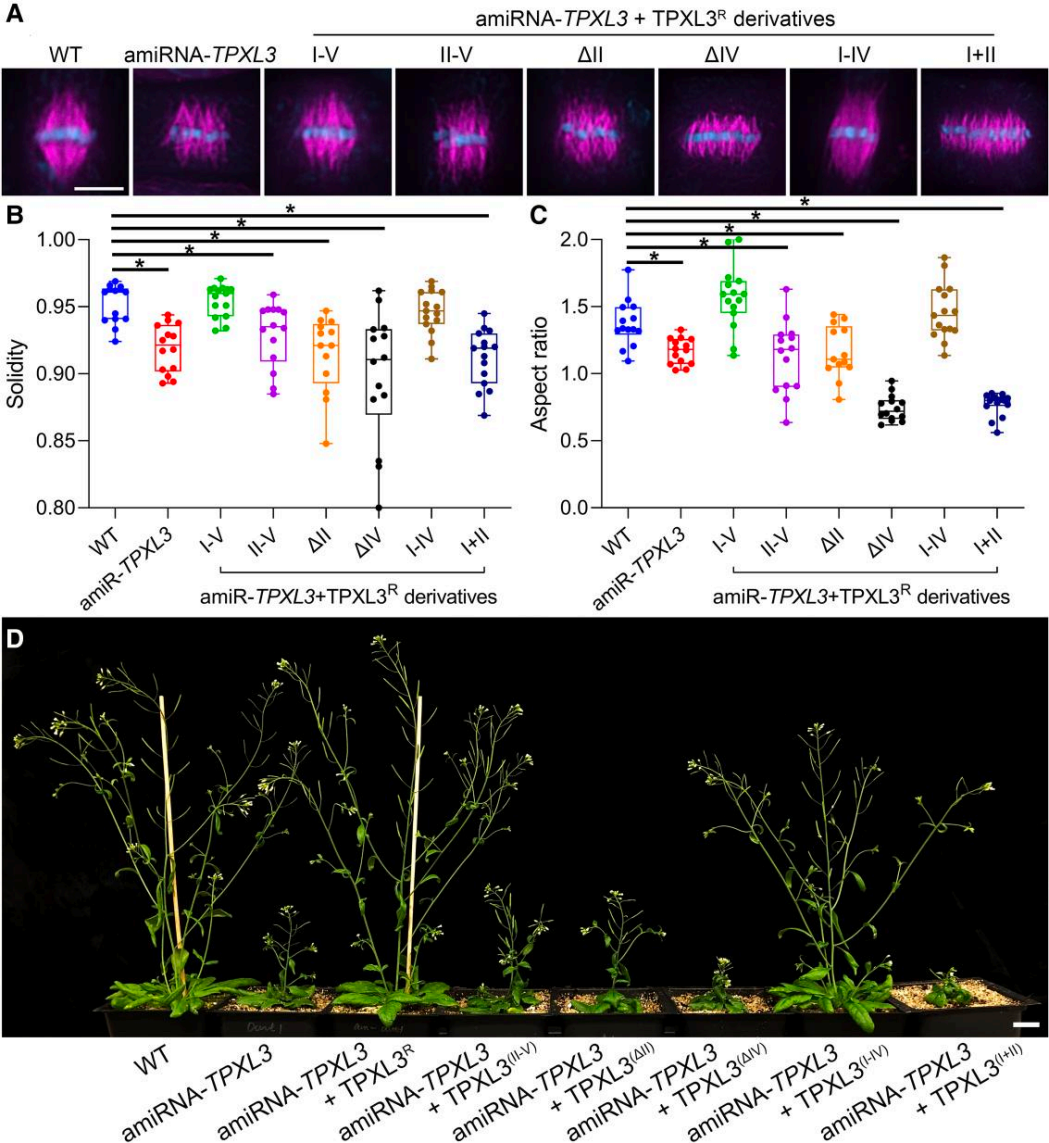
Compromised spindle apparatus is associated with retarded plant growth. **A)** Spindle microtubule arrays in metaphase cells indicated by the aligned chromosomes at the equatorial plane from indicated transgenic lines. While the amiRNA-*TPXL3* mutant cell produces a spindle with disorganized spindle poles, the expression of the full-length microRNA-resistant TPXL3^R^ restores the spindle morphology. So is the truncated protein lacking C-terminal domain (TPXL3^I−IV^). While the deletion of domains I (TPXL3^II−V^) or II (TPXL3^ΔII^) does not render a toxic effect on the amiRNA-*TPXL3* spindles, the deletion of domain IV (TPXL3^ΔIV^) or III–V (TPXL3^I−II^) greatly enhanced the deformation of spindles that have kinetochore MT fibers arranged in parallel bundles. The micrographs represent metaphase cells of root tips of at least 20 seedlings in each of 2 independent transgenic events for every construct, which show consistent results observed in both transgenic lines. **B)** The mean solidity of spindles is reduced to various degrees in the mutants expressing TPXL3 derivatives missing corresponding domain(s) when compared to the control cells by using cells processed for anti-tubulin immunofluorescence. The statistical significance of the difference between the amiR-*TPXL3* cells (*n* = 14) or amiR-*TPXL3* cells expressing microRNA-resistant forms of TPXL3 derivatives (I–V, *n* = 15; II–V, *n* = 14; ΔII, *n* = 13; ΔIV, *n* = 14; I–IV, *n* = 15; or I + II, *n* = 15) and the wild-type (*n* = 15) control cells is assessed by the *U* tests that result in averages ± standard deviations of 0.952 ± 0.015, 0.919 ± 0.018, 0.955 ± 0.012, 0.929 ± 0.024, 0.915 ± 0.029, 0.898 ± 0.051, 0.946 ± 0.016, and 0.912 ± 0.021 with *P* values of 3.575e-05, 0.722, 0.009383, 5.319e-05, 0.0001201, 0.3468, 3.288e-06, respectively. Asterisks indicate significant differences. **C)** The mean aspect ratio of spindles formed in mutant cells expressing TPXL3 derivatives missing corresponding domain(s) is reduced to various severities when compared to the control cells. The statistical significance of the difference between the amiR-*TPXL3* cells or amiR-*TPXL3* cells expressing microRNA-resistant forms of TPXL3 derivatives (I–V, II–V, ΔII, ΔIV, I–IV, or I + II) and the wild-type control cells is assessed by the U tests that result in averages ± standard deviations of 1.344 ± 0.172, 1.168 ± 0.097, 1.571 ± 0.240, 1.136 ± 0.290, 1.162 ± 0.197, 0.743 ± 0.097, 1.459 ± 0.208, and 0.772 ± 0.085 with *P* values of 0.0005233, 0.01202, 0.006743, 0.007796, 4.985e-08, 0.2752, 2.579e-08, respectively. Asterisks indicate significant differences. **D)** Growth phenotypes associated with the expression of various TPXL3 derivatives. While the expression of microRNA-resistant TPXL3^R^ greatly suppresses the growth phenotype of the amiRNA-*TPXL3* plant, the expression of TPXL3^II−V^ or TPXL3^ΔII^ does not significantly alter the growth of the mutant. However, the expression of TPXL3^ΔIV^ or TPXL3^I−II^ further enhances growth defects as indicated by severe inhibition of axial growth and production of inflorescence. Representative plants are randomly selected from 5 independent transgenic lines that have been chosen from ten or more exhibiting consistent phenotypic characteristics for each construct. Data as individual points are presented in box-and-whisker plots, showing the interquartile range (box), the median (horizontal line), and minimum and maximum values (whiskers). Scale bars, 5 *µ*m (**A**, applicable to all micrographs included) and 2 cm (**B**).

To explore the functionality of different TPXL3 domains in spindle morphogenesis, we expressed different derivatives of TPXL3 under the control of the native TPXL3 promoter in the amiR-*TPXL3* mutant plants. The amiR-*TPXL3* (line 5) mutant line is the host for the expression of different TPXL3 derivatives. First, we used anti-tubulin immunofluorescence to examine metaphase spindle microtubule arrays and detected disorganized poles in mutant cells, similar to what was observed by live-cell imaging (Fig. 7A). Again, the morphological changes of mitotic spindles were also assessed quantitatively by determining their solidity and aspect ratio values according to the method described earlier (Fig. 7B, Supplementary Fig. S8). When the full-length microRNA-resistant form of TPXL3 (TPXL3^R^) was expressed, metaphase cells restored typical spindles with converged poles (Fig. 7A). Concomitantly, the transgene suppressed the stunted growth phenotype of the host plant and rendered adult plants that resembled the wild-type control (Fig. 7D). Then, we had the AUR-binding motif (domain I) or the microtubule-binding site (domain II) removed and found that the truncated TPXL3^II−V^ and TPXL3^ΔII^ proteins did not restore the spindle morphology, neither did they improve seedling growth (Fig. 7, A and D, Supplementary Fig. S10). These results supported the notion that the TPXL3 function was inseparable from its interactions with α Aurora or microtubules. Surprisingly, the TPXL3^I−IV^ derivative with the C-terminal domain V removed was able to restore the converged spindle microtubule arrays in metaphase cells and growth and reproduction almost as robust as the wild-type control (Fig. 7, A and D, Supplementary Fig. S10). The derivative with domain IV removed, however, enhanced spindle defects with kinetochore-microtubule fibers arranged in a palisade-like fashion and resulted in great retardation of plant growth (Fig. 7, A and D, Supplementary Fig. S10). Similarly, expression of a truncated version of TPXL3 containing only domains I and II resulted in a similar if not more exaggerated negative impact in both spindle morphogenesis and seedling growth as TPXL3^I−IV^ (Fig. 7, A and D, Supplementary Fig. S10). Such exaggeration of spindle defects was clearly expressed in the reductions of both the solidarity and aspect ratio values (Fig. 7, B and C). The highest reductions of both solidarity and aspect ratio were detected in cells of the mutant plants expressing TPXL3^ΔIV^ or TPXL3^I + II^ that also showed the most severe seedling growth defects.

Collectively, these results affirmed that defects in spindle microtubule organization are translated into deficiencies in overall growth besides demonstrating the indispensability of domains I (AUR-binding), II (microtubule-binding), and IV (importin binding) for TPXL3 function.

## Discussion

The dissection of the essential TPXL3 function indicated that TPXL3 not only plays an essential role in targeting α Aurora to spindle microtubules and activating its kinase activity but also governs the localization of the microtubule-nucleating factor γ-tubulin complex. TPXL3 is required for spindle morphogenesis that directly contributes to robust axial growth in *A*. *thaliana*.

### The TPXL3 but not TPX2-regulated function for α Aurora is essential in *A*. *thaliana*

The evolutionarily conserved TPX2 protein, first identified as an interacting/targeting protein of the Kinesin-12 motor XKLP2 in frog cells, is one of the most visible and important microtubule-associated proteins (MAP) for mitotic spindle assembly (Petry 2016). The utmost noticeable function of TPX2 is to target Aurora A to spindle microtubules toward spindle poles in order to phosphorylate proteins like microtubule-based motors and MAPs that often play critical roles in the organization of spindle poles (Meunier and Vernos 2016). Recently, TPX2 was also identified as a microtubule-nucleating factor that works with the augmin complex for activating the γ-tubulin ring complex in order to produce branching microtubules on the wall of extant microtubules (Alfaro-Aco et al. 2020). TPX2 interacts with importin and is released when importin binds to Ran-GTP generated by the RanGEF RCC1 associated with chromatin (Meunier and Vernos 2016). TPX2 interacts with motors of Kinesin-5 and Kinesin-12 for their localization and spindle pole organization (Wittmann et al. 2000; Ma et al. 2010, 2011). These features determine how important TPX2 is for spindle assembly.

*A. thaliana*, representing flowering plants, produces a single TPX2 and 8 TPXL proteins (Tomastikova et al. 2015). Among the 8 TPXL proteins, 5 contain the Aurora-binding motif so that perhaps their functions all are linked to the Aurora kinase (Smertenko et al. 2021). When co-expressed, these TPX2-like proteins exhibit different subcellular localizations, suggesting functional diversification (Dvorak Tomastikova et al. 2020). Because TPXL proteins all miss one or more domains found in the canonical TPX2, TPX2 could have had the most versatile or perhaps most critical function in mitosis. Indeed, AtTPX2 not only exhibits a typical spindle pole-biased localization pattern but also is competent to induce ectopic microtubule production upon over-expression (Petrovska et al. 2013; Boruc et al. 2019). Therefore, it was surprising to discover that in *A*. *thaliana* TPXL3 becomes essential for mitosis while the canonical TPX2 is dispensable although both interact with the α Aurora kinase in vivo (Boruc et al. 2019). When considering that TPXL3 misses the extended C-terminal domain that accounts for kinesin interaction, one would expect that the canonical TPX2 possesses functions that are likely missing in TPXL3. This phenomenon may be attributed to the specification of substrates brought about by the difference between the two proteins. Besides TPX2, the Kinesin-5 motor Eg5, Polo-like kinase, CDK5RAP2, and the TACC (transforming acidic coiled-coil) protein are among the most noticeable or perhaps most important substrates of the vertebrate TPX2 (Barr and Gergely 2007; Magnaghi-Jaulin et al. 2019). In plants, there are no obvious homologs of Polo, CDK5RAP2, or TACC (Yamada and Goshima 2017). Although a temperature-sensitive mutation in a Kinesin-5 gene *RSW7* causes spindle collapse at restrictive temperatures, T-DNA insertional mutations do not seem to cause a noticeable phenotype (Bannigan et al. 2007; Gillmor et al. 2016). Therefore, if AtTPX2 functions in the regulation of Kinesin-5 in *A*. *thaliana*, such a function assigned to TPX2 may not be essential, just like RSW7 being dispensable. Coincidently, among TPX2 family proteins of animal origins, the TPX2 homolog in Drosophila Mei-38 lacks both the Aurora-binding site and the C-terminal Kinesin-5-binding domain and is dispensable for spindle assembly during meiosis and mitosis (Wu et al. 2008; Goshima 2011). Therefore, plants and Drosophila and perhaps other insects have their essential function of Aurora kinase independent of the TPX2 proteins.

The notion that TPXL3 was not the sole activator of α Aurora is also supported by a different localization of the 2 proteins during cytokinesis. At late stages of cytokinesis, α Aurora can be detected in the phragmoplast midzone (Van Damme et al. 2004). This localization is important for downregulation of microtubule-associated protein MAP65-3 association with midzone microtubules for phragmoplast expansion (Deng et al. 2024). However, TPXL3 was only detected at the distal ends of the phragmoplast, facing the re-forming daughter nuclei, but not in the phragmoplast midzone. Therefore, other TPX2-like proteins must have targeted the kinase there, and such a role awaits the investigation of uninvestigated candidates.

Conversely, TPXL3 likely bears features that are not shared by TPX2, e.g. specifying selective substrates that are not recognized by TPX2. Based on the phenotype of compromised localization of the γTuRC, it is likely that certain subunits of the complex or its regulatory proteins may be substrates of α Aurora-TPXL3. Proteomic experiments show that most γTuRC subunits (especially GCP6) and its targeting factor NEDD1 are heavily phosphorylated in vivo (Teixido-Travesa et al. 2012). In animal cells, downregulation of Aurora function leads to a decrease or loss of conspicuous association of NEDD1 with mitotic microtubule arrays, indicating regulation of NEDD1 activity by the kinase (Pinyol et al. 2013; Courtheoux et al. 2019). Recently, we showed that in *A*. *thaliana* GCP6 also plays a critical role in the spindle localization of γ-tubulin (Miao et al. 2019). It would be interesting to test whether AtGCP6 shows a cell cycle-dependent phosphorylation pattern linked to its function in spindle morphogenesis. Studies in vertebrates also identified the MAP subunit of the augmin complex as a substrate of Aurora A and showed the phosphorylation downregulate its microtubule binding and spindle localization (Tsai et al. 2011). Although plants also produce the 8-subunit augmin complex, the polypeptide sequences of its subunits often are very divergent from the animal counterparts (Ho et al. 2011; Hotta et al. 2012; Nakaoka et al. 2012). In *A*. *thaliana*, EDE1 serves as the M phase-specific microtubule-associated protein (MAP) subunit of augmin and is required for the complex to decorate spindle microtubule array (Lee et al. 2017). Again, it remains to be tested whether EDE1 is recognized by the α Aurora-TPXL3 complex and has its function regulated by Aurora phosphorylation.

On the other hand, TPXL3 exhibited an intriguing localization pattern toward the later stages of cytokinesis. Its fluorescent signal left wide gaps in the middle zone of the phragmoplast and it was absent from the bulk of phragmoplast microtubules while being prominent at the distal ends of the phragmoplast facing daughter nuclei. This is different from the localization pattern of the microtubule nucleator γ-tubulin which shows a biased distribution pattern with a prominent association with minus ends of phragmoplast microtubules at all stages. As shown here, the dark zone left the fluorescent TPXL3 signal was obviously wider than that of γ-tubulin, suggesting that it might not be directly decorating phragmoplast microtubule minus ends like γ-tubulin. It would be interesting to test whether such a highly polarized localization pattern was dependent on dynamic microtubules. Further investigations of TPXL3-interacting proteins by sensitive assays could lead to identifications of factors that interact directly or indirectly (e.g. *via* importin) with TPXL3 and act together for cell cycle-dependent events.

### TPXL3-dependent spindle morphogenesis

Angiosperms have lost the centriole-based centrosome structure so that their cells form acentrosomal spindle microtubule arrays during mitosis, unlike fungal and typical animal cells that have spindle poles focused on the centrosomes. However, mitotic plant cells have spindle microtubules converged toward spindle poles despite the lack of the centrosome structure (Bajer and Mole-Bajer 1986; Bajer et al. 1993). These so-called microtubule-converging centers are likely established by minus end-directed motors (She and Yang 2017). In animal cells, spindle pole focusing is largely brought about by the microtubule minus end-directed motor cytoplasmic dynein together with assistance from Kinesin-14 motors (Heald and Khodjakov 2015; Guilloux and Gibeaux 2020). Plants lack cytoplasmic dynein and produce an expanded subfamily of Kinesin-14 motors with 21 members in *A*. *thaliana* that are predicted to be minus end-directed (Reddy and Day 2001). Among them, the KatA/ATK1 and ATK5 perhaps play a dominant role in spindle morphogenesis as the loss of either motor leads to mitotic spindles with widened poles (Liu et al. 1996; Marcus et al. 2003; Ambrose et al. 2005; Hotta et al. 2022). Single *atk1* or *atk5* mutant does not exhibit obvious vegetative growth phenotype but the simultaneous loss of both leads to lethality (Quan et al. 2008), suggesting that enhanced challenges to spindle morphogenesis may have caused spindle malfunctioning. The question is whether KatA/ATK1 and ATK5 are substrates of α Aurora-TPXL3 and their functions in spindle assembly are regulated by phosphorylation. In other words, the next step would be to test whether the function of TPXL3 in spindle assembly is mediated by KatA/ATK1 and ATK5. This task is challenged by the lethality of the *atk1 atk5* double mutant so a conditional mutation would be useful.

On the other hand, defects in spindle microtubule arrays in the amiR-*TPXL3* cells might be caused by lack of γTuRC activity associated with spindle microtubules. The vertebrate Aurora A-TPX2 complex recruits the γTuRC to spindle poles by a direct interaction between TPX2 and a protein called XHRAMM which in turn brings in the γTuRC-targeting factor NEDD1 (Pinyol et al. 2013). However, there is no obvious homolog of XHRAMM in angiosperms based on amino acid sequence comparison. Therefore, plants must have evolved a different mechanism that regulates the activity of γTuRC on spindles, especially for pole organization.

It is often enigmatic how morphologically defective spindles might affect vegetative growth or whether the spindle pole convergence is linked to plant wellness. Although defects in cell elongation could also compromise axial growth, the dwarf growth phenotype exhibited by various *tpxl3* mutants was unlikely caused by an interphase-specific defect based on the following arguments. First, the *TPXL3* gene shows specific expression in dividing meristematic cells by single-cell transcriptomic analysis (Ryu et al. 2019). Second, TPXL3 is a nuclear protein in interphase cells so that it would not interact with cortical microtubules required for cell elongation. Third, the amiR-*TPXL3* mutant cells established cortical microtubule arrays indistinguishable from those in the wild-type cells. The linked phenotypes summarized here provide direct evidence showing that morphologically compromised spindles significantly affected axial growth because of the reduced function of α Aurora and TPXL3. Our results demonstrated that the more severely detorted spindles were associated with more retarded axial growth. Therefore, the α Aurora/TPXL3-associated mitotic function is an important factor in promoting robust vegetative growth in *A*. *thaliana* exampling angiosperms.

## Materials and methods

### Plant materials, transformation, and growth conditions

Mutant seeds of *A. thaliana* are obtained from the Arabidopsis Biological Research Center (ABRC). These include the SAIL_350_B08 and GABI_480B12 lines for the *TPXL3* (AT4G22860) locus, SALK_079098 for the *TPXL2* (At4g11990) locus, and the *aur1-2/aur2-2* mutant (Van Damme et al. 2011; Boruc et al. 2019). All plants were grown under a 16-h-light and 8-h-dark cycle with 70% relative humidity at 22 ℃. Live-cell imaging and immunolocalization experiments were carried out using young seedlings germinated on a solid medium supplied with 1/2 Murashige Skoog salt mixture (Sigma).

Transformation of *A*. *thaliana* plants was carried out by floral dipping and using the *Agrobacterium tumefacien* strain GV3101. Among transformants recovered by antibiotic or herbicide selection, at least 10 that showed consistent growth phenotypes were kept for seed production in each transformation experiment. Among 3 independent lines that showed consistent results in phenotypic and microscopic examination, one of them was used in later experiments. Among transgenic plants examined in this study, independent lines from each transformation experiment rendered identical results. *A*. *thaliana* root cells were observed 4 days after germination.

### Plasmid construction

Primers used in this study and their corresponding sequences are listed in Supplementary Table S1. Genomic fragments of TPXL2, TPXL3, and AUR1, which contain the promoter and coding sequences, were ampliﬁed using Phusion DNA polymerase (ThermoFisher Scientific, catalog # F549S). The amplified fragments were cloned into the Gateway pENTR/D-TOPO vector (ThermoFisher Scientific, catalog # K240020SP). To produce the GFP-AUR1 or Flag-AUR1 fusion construct, the entry vector containing genomic AUR1 was linearized by inverse PCR, and then, an EGFP or Flag fragment was inserted in front of the start codon via the Gibson Assembly method. The resulting plasmids were recombined with pGWB4 or pGWB10 (Nakagawa et al. 2007) by recombination reactions with LR clonase (ThermoFisher Scientific, catalog # 11791100).

Constructs for *N*. *benthamiana* leaf inﬁltration experiments were produced as follows. The cDNA fragments of *TPXL3* or *AUR1* were amplified using the RAFL07-16-B14 or RAFL07-91-B16 plasmids (RIKEN, Japan) as the templates. The resulting products were cloned into the Gateway pENTR/D-TOPO vector. The entry vector containing TPXL3 CDS was linearized by reverse PCR and was ligated using a *BamH*I site to produce truncated TPXL3 vectors. The resulting entry clones were delivered into the destination vector pGWB605 or pGWB661 (Nakamura et al. 2010). The microtubule marker CKL6-mCherry was expressed by using the construct as reported (Ben-Nissan et al. 2008).

### amiR-*TPXL3* construction and complementation

The artiﬁcial miRNA construct was designed according to published protocols (Carbonell et al. 2014). Brieﬂy, the amiR-TPXL3 guide sequence was designed *via* P-SAMS (http://p-sams.carringtonlab.org/amirna/designer). The target sequence of 5′-CGGAAAAAGAGCACTCCGAAA-3′ has 3 mismatches with the corresponding sequence in TPXL2 (5′-CGAAAAAACAGCACTCCTAAA-3′) but not other TPX2 family genes. Because five or less mismatches are required for the silencing effect (Schwab et al. 2006), amiR-*TPXL3* was unlikely to target *TPXL2*. The guide DNA fragment was cloned into the pENTR-AtMIR390a vector (Carbonell et al. 2014), followed by an LR reaction with the pGWB602Ω destination vector (Nakamura et al. 2010). The resulting amiR-TPXL3 plasmid was introduced into *Arabidopsis* wild-type (Col) plants and transgenic T1 plants were selected by spraying Finale (AgrEvo) for Basta resistance. To analyze the silencing efficiency, RNA samples were prepared from young seedlings of 3-day-old amiR-*TPXL3* lines and wild-type control plants, followed by real-time quantitative RT-PCR according to a previous study (Miao et al. 2019).

To make a construct that contains an amiR-*TPXL3*-resistant version of *TPXL3*, 8 silent mutations were introduced into the TPXL3 entry clone using the Gibson Assembly method. Speciﬁcally, the amiR-TPXL3 target site 5′-CGGAAAAAGAGCACTCCGAAA-3′ was replaced by 5′-CGtAAgAAatcCACaCCaAAg-3′, with base changes indicated by lowercase letters. This entry clone was introduced into a binary vector pGWB4 for generating a GFP fusion by the LR reaction and transformed into the amiR-*TPXL3* lines #5 and #3.

### Transient expression in *Nicotiana benthamiana*

*Agrobacterium* GV3101 carrying each constructed plasmid was cultured overnight at 28 °C and resuspended in an infiltration buffer containing 10 mm MES (pH 5.7), 10 mm MgCl_2_ and 150 *μ*M acetosyringone to a final OD600 of 1.0, and equal volumes of cultures carrying different constructs were mixed for co-inﬁltration. These cells were then mixed with *Agrobacterium* C58C1 (pCH32-35S:p19) in a 1:1 ratio. After incubation for 3 h at room temperature, the resulting cultures were infiltrated into the leaves of 4-week-old *N. benthamiana* using a syringe. Localization of expressed proteins was observed under fluorescence microscopy 3 days after infiltration. Leaf cells were induced to enter mitosis by ectopic expression of Cyclin D as described in the established CDELS protocol (Xu et al. 2020).

### Production of recombinant proteins and in vitro kinase assays

The coding sequences of TPXL3 or AUR1 were cloned into pGEX-KG (Guan and Dixon 1991) at the *EcoR*I and *Nco*I sites or pET28a at the *BamH*I and *Sal*I sites. The recombinant plasmids rendered the expression of GST or His-tagged proteins in bacteria host BL21. The fusion proteins were puriﬁed using Glutathione HiCap matrix (Qiagen, catalog # 30900) or Ni-NTA agarose resins (Yeasen, catalog # 20502ES) according to the manufacturer's instructions. For the pull-down assay, GST-fused TPXL3 or TPXL3 truncations and His-fused AUR1 were mixed with glutathione beads and incubated at 4 °C for 2 h. Beads were washed 5 times, and proteins were eluted from beads by boiling in an SDS loading buffer and then separated by SDS-PAGE.

For in vitro kinase assay, GST, GST-TPXL3, and truncated GST-TPXL3 were incubated with GST-AUR1 in 40 *μ*L of kinase buffer [10 mm HEPES (pH 7.5), 20 mm MgCl_2_, 1 mm DTT, 5 mm EGTA, 0.1 mm ATP]. After incubation at 37 °C for 60 min, the reactions were stopped by adding 10 *μ*L of 5×SDS loading buffer and boiling for 10 min. Proteins were resolved by SDS-PAGE and phosphorylation was detected by the Pro-Q Diamond Phosphoprotein Gel Staining Kit (ThermoFisher Scientific, catalog # P33301).

### Immunolocalization and fluorescence microscopy

For immunolocalization, meristematic cells collected from 20 or more roots were used according to previously described protocols (Lee and Liu 2000). Experiments were repeated by using seeds collected from at least one more independent transformant. The primary antibodies used here include the rabbit anti-GFP polyclonal antibody (1:400, ThermoFisher, catalog # A-6455), DM1A mouse anti-α-tubulin monoclonal antibody (1:400, Sigma-Aldrich, catalog # T9026), sheep anti-tubulin polyclonal antibodies (1:400, Cytoskeleton, catalog # ATN02), G9 mouse anti-γ-tubulin monoclonal antibody (1:400, Horio et al. 1999), and C2 mouse anti-FLAG monoclonal antibody (1:400, Shanghai Genomics, catalog # GNI4310-FG). Secondary antibodies are fluorescein isothiocyanate (FITC)-conjugated donkey anti-rabbit IgG, FITC-conjugated donkey anti-mouse IgG, and Texas Red-conjugated donkey anti-mouse IgG (1:400, Rockland Immunochemicals, catalog # 611-702-127, 610-702-124, and 610-709-124). Stained cells were observed under an Eclipse 600 microscope equipped with 60× Plan-Apo and 100× Plan-Fluor objectives (Nikon) and coupled with filter sets of GFP BrightLine (Semrock, catalog # GFP-3035B-NQF-ZERO), Texas Red BrightLine (Semrock, catalog # TXRED-4040B-NQF-ZERO), and DAPI BrightLine (Semrock, catalog # DAPI-5060B-NQF-ZERO). Images were acquired by an OptiMOS camera (Q Imaging) controlled by the µManager software package.

For live-cell observation, 5-day-old seedlings were transferred to glass slides and submerged in water. Root meristematic cells were observed under an Axio Observer inverted microscope equipped with the LSM710 laser scanning confocal module (Carl Zeiss) by using a 40× C-Plan (water) objective. The GFP and mCherry signals were excited by using 488 and 561-nm lasers, respectively, at 10% power level and collected by using the manufacturer's standard settings for the respective fluorescent proteins with a manufacture-defined master gain value of 700. Images were acquired using the ZEN software package (Carl Zeiss) and processed in ImageJ (https://imagej.net/ij/).

### Image processing to quantify spindle morphology

To quantitatively evaluate spindle morphology, we first obtain binary images from the time-lapse confocal images by Gaussian filter (Sigma = 2 pixels) and Otsu's thresholding (Supplementary Fig. S8A). From the binary images, the segmented spindle solidity and aspect ratio were analyzed using the “Analyze Particles' command in the ImageJ software” (Schneider et al. 2012; Higaki et al. 2017). To quantify the spindle morphology using anti-tubulin immunolocalization data, the fluorescence images were binarized by Otsu's thresholding followed by analysis by using ImageJ as described for the confocal images.

### Statistical analysis

Mann–Whitney *U* test was used for comparisons between cells of different genotypes, as described in the figure legends. Quantifications are reported as mean ± standard deviation in the figure legends. Other statistical details are explained in the figure legends.

### Accession numbers

Sequence data from this study can be found in The Arabidopsis Information Resource (www.arabidopsis.org) under the following accession numbers: TPXL3, AT4G22860; TPXL2, AT4G11990; and AUR1, AT4G32830.

## Supplementary Material

koaf065_Supplementary_Data

## Data Availability

All data acquired from this study are included within the main text and Supplementary information.

## Supplementary data

The following materials are available in the online version of this article.

**Supplementary Figure S1.** Suppression of amiRNA-*TPXL3* by expressing a microRNA-resistant *TPXL3*.

**Supplementary Figure S2.** Organization of cortical microtubules in the wild-type control, amiR-*TPXL3*, and amiR-*AUG6* cells.

**Supplementary Figure S3.** TPXL3, but not TPXL2, is associated with mitotic spindles.

**Supplementary Figure S4.**  *AUR1* expression rescues the *aur1 aur2* double mutant growth defect.

**Supplementary Figure S5.** TPXL3 localization is not altered in the *aur1 aur2* double mutant.

**Supplementary Figure S6.** Detection of GFP-AUR1 in control and amiR-*TPXL3* root cells.

**Supplementary Figure S7.** Interaction between TPXL3 and AUR1 in a pull-down assay.

**Supplementary Figure S8.** Image processing and quantification of the spindle morphology.

**Supplementary Figure S9.** The mitotic spindles in the amiR-*TPXL3* cells are hypersensitive to oryzalin.

**Supplementary Figure S10.** Quantitative assessment of the root growth phenotypes associated with the expression of the TPXL3 derivatives.

**Supplementary Table S1.** Primers used in this study.

**Supplementary Video S1.** Dynamic redistribution of TPXL3-GFP during mitosis.

**Supplementary Video S2.** A wild-type control cell assembles mitotic microtubule arrays from prophase to cytokinesis. Microtubules are marked by a VisGreen-TUB6 fusion protein.

**Supplementary Video S3.** An amiRNA-*TPXL3* mutant cell exhibits disorganized microtubule arrays during mitosis when compared to the control. Microtubules are marked by a VisGreen-TUB6 fusion protein.

**Supplementary Video S4.** Formation of bipolar spindle microtubule arrays in a control cell in the presence of 100 nm oryzalin.

**Supplementary Video S5.** Disorganized microtubule bundles are formed in an amiR-*TPXL3* cell in the presence of 100 nm oryzalin.

## Dive Curated Terms

The following phenotypic, genotypic, and functional terms are of significance to the work described in this paper:

NEDD1 Gramene: AT5G05970
NEDD1 Araport: AT5G05970

ATK5 Gramene: AT4G05190
ATK5 Araport: AT4G05190

EDE1 Gramene: AT2G44190
EDE1 Araport: AT2G44190

ATK1 Gramene: AT4G21270
ATK1 Araport: AT4G21270

GCP6 Gramene: At3g43610
GCP6 Araport: At3g43610

AUR2 Gramene: At2g25880
AUR2 Araport: At2g25880

AUR1 Gramene: At4g32830
AUR1 Araport: At4g32830
