# Supplementary material for: The unconventional TPX2 family protein TPXL3 regulates α Aurora kinase function in spindle morphogenesis in Arabidopsis
Source: Plant Cell. 2025 Mar 26;37(4):koaf065. doi: 10.1093/plcell/koaf065 (PMC12012799; doi:10.1093/plcell/koaf065)
Supplement: koaf065_Supplementary_Data [file koaf065_supplementary_data.zip › Deng2025R_Supplemental Figures_0308.pdf]

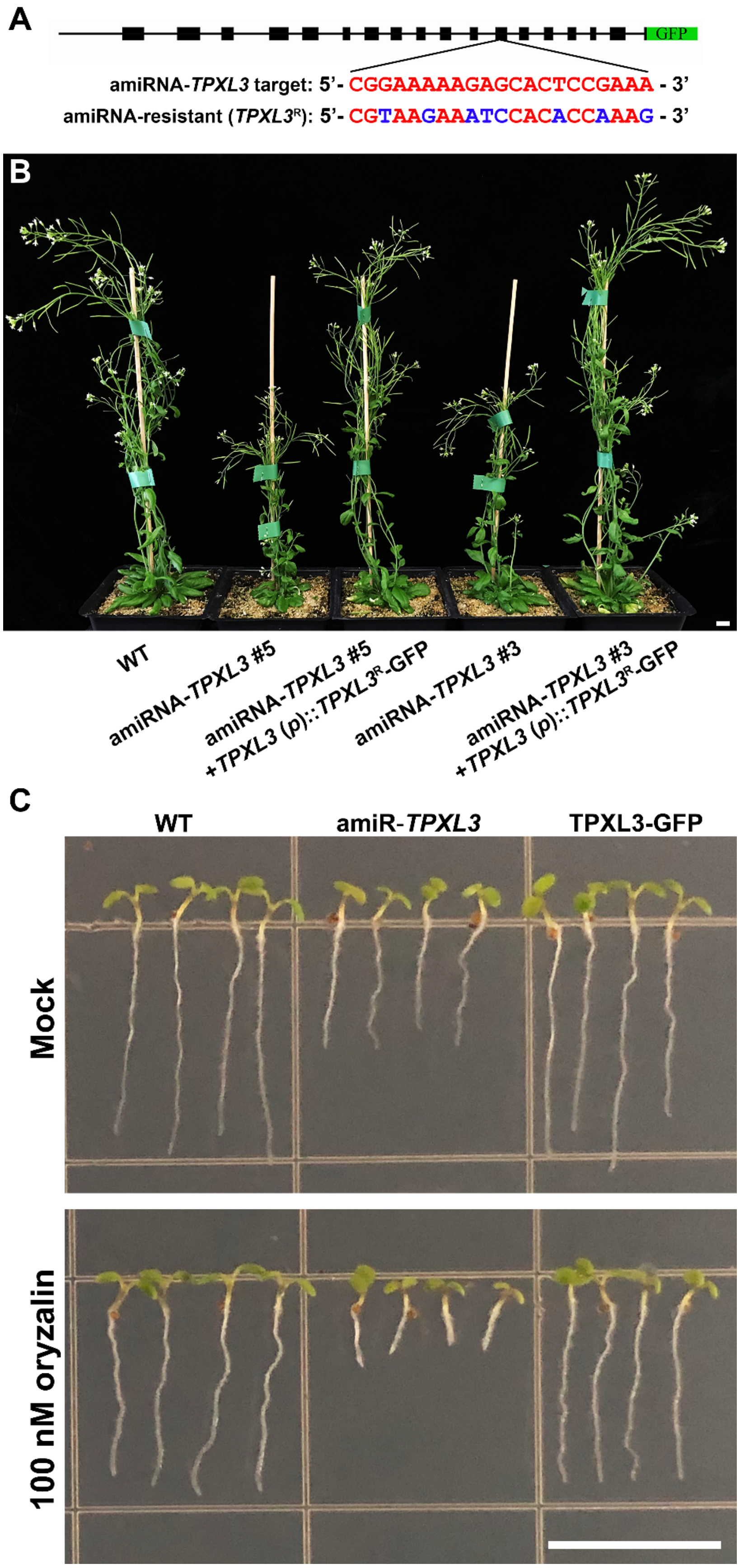
A
GFP
amiRNA-TPXL3 target: 5'- CGGAAAAAGAGCACTCCGAAA - 3'
amiRNA-resistant (TPXL3R): 5'- CGTAAGAAATCCACACCAAAG - 3'
B
WT
amiRNA-TPXL3 #5
amiRNA-TPXL3 #5 +TPXL3 (p)::TPXL3R-GFP
amiRNA-TPXL3 #3
amiRNA-TPXL3 #3 +TPXL3 (p)::TPXL3R-GFP
C
WT
amiR-TPXL3
TPXL3-GFP
Mock
100 nM oryzalin

Supplemental Figure S1. Suppression of amiRNA-*TPXL3* by expressing a microRNA-resistant *TPXL3***.** (**A**) The target of amiRNA-*TPXL3* and silent mutations in the *TPXL3*^{R} gene. Silent mutations in the TPXL3 gene are aimed at resistance to amiR-*TPXL3*. (**B**) The amiRNA-*TPXL3* challenges are suppressed by the microRNA-resistant form of TPXL3, as reflected by the restoration of seedling growth upon the expression of TPXL3^{R} in two independent amiRNA-*TPXL3* lines. (**C**) Control (WT), amiR-*TPXL3*, and amiR-*TPXL3* expressing microRNA resistant TPXL3^{R}-GFP seedlings were grown on mock and 100 nM oryzalin media for seven days. Only the amiR-*TPXL3* seedlings have root growth greatly compromised. The amiR-*TPXL3* seedlings show retarded root growth and hypersensitivity to 100 nM oryzalin. Scale bars (B and C), 1 cm.

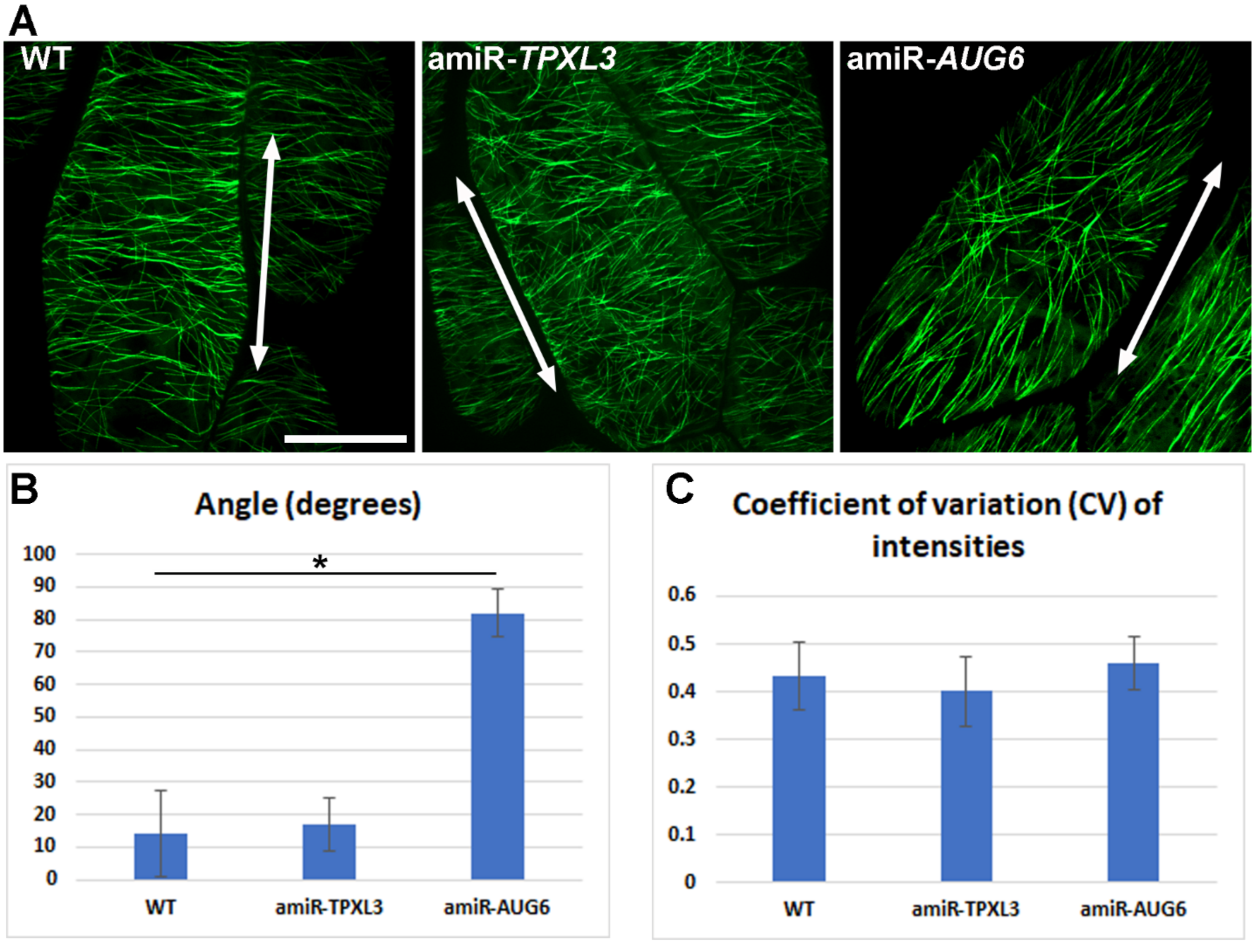

Supplemental Figure S2. Organization of cortical microtubules in the wild-type (WT) control, amiR-*TPXL3*, and amiR-*AUG6* cells that express the VisGreen-TUB6 fusion protein. The amiR-*AUG6* serves as a reference. (**A**) The amiR-*TPXL3* cells form cortical microtubule arrays that are largely in the transverse orientation perpendicular to the cell elongation axis (double arrows), similar to those in the WT cells; but the amiR-*AUG6* mutant cells assemble cortical microtubules often along the cell elongation axis. (**B**) Quantitative assessment of cortical microtubule orientation with 0 degree reflecting transverse orientation. The mean values and S.D. are 14.36±13.21 (WT, n=10), 16.76±8.20 (amiR-TPXL3, n=9), and 82.04±7.53 (amiR-*AUG6*, n=7). The difference between WT and amiR-*TPXL3* is not significant (p-value = 0.447) but that between WT and amiR-*AUG6* is (p-value = 0.0001028) is as indicated by the asterisk. (**C**) Quantitative assessment of microtubule bundling reflected by coefficient of variation (CV) of intensities. The CV values and S.D. are 0.43±0.07 (WT), 0.40±0.07 (amiR-*TPXL3*), and 0.46±0.06 (amiR-*AUG6*). The differences between WT and amiR-*TPXL3* (p-value = 0.4002) and between WT and amiR-*AUG6* (p-value = 0.4747) are not significant. Scale bar, 10 µm.

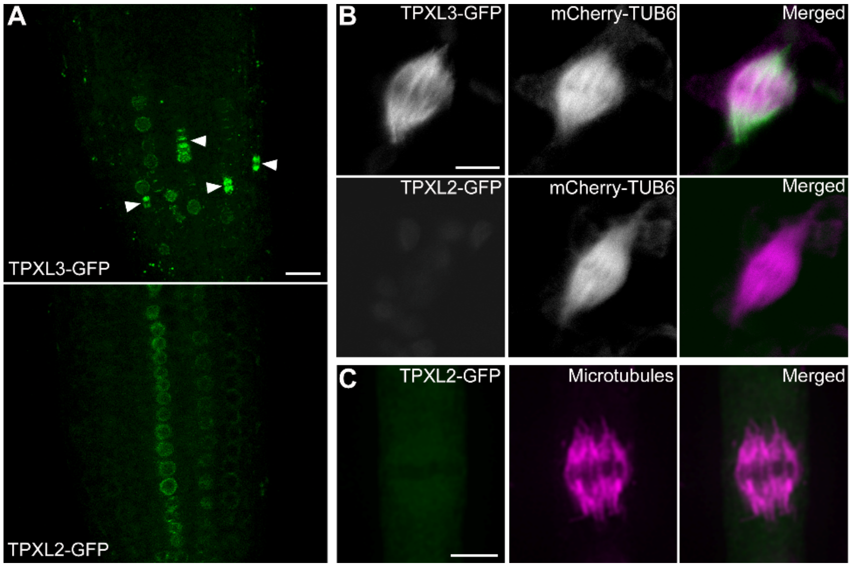

Supplemental Figure S3. TPXL3 but not TPXL2 is associated with mitotic spindles. (**A**) Root cells express TPXL3-GFP (top) or TPXL2-GFP (bottom) under the control of their native promoters. Conspicuous TPXL3 signal shows spindle like patterns (arrowheads) while TPXL2 is detected in nuclei only. (**B**) When expressed in tobacco cells induced into mitosis, TPXL3-GFP decorates spindle microtubules marked by mCherry-TUB6 and is biased towards spindle poles. In contract, TPXL2-GFP is undetectable on spindle microtubules. (**C**) Localization of the TPXL2-GFP fusion protein by immunofluorescence in the transgenic Arabidopsis line expressing TPXL2-GFP. A metaphase cell establishes a bipolar spindle with chromosomes aligned in the middle. TPXL2-GFP is detected as evenly diffuse signal in the cytoplasm. Scale bars (applicable to all micrographs), 20 µm (**A**), 5 µm (**B**, **C**).

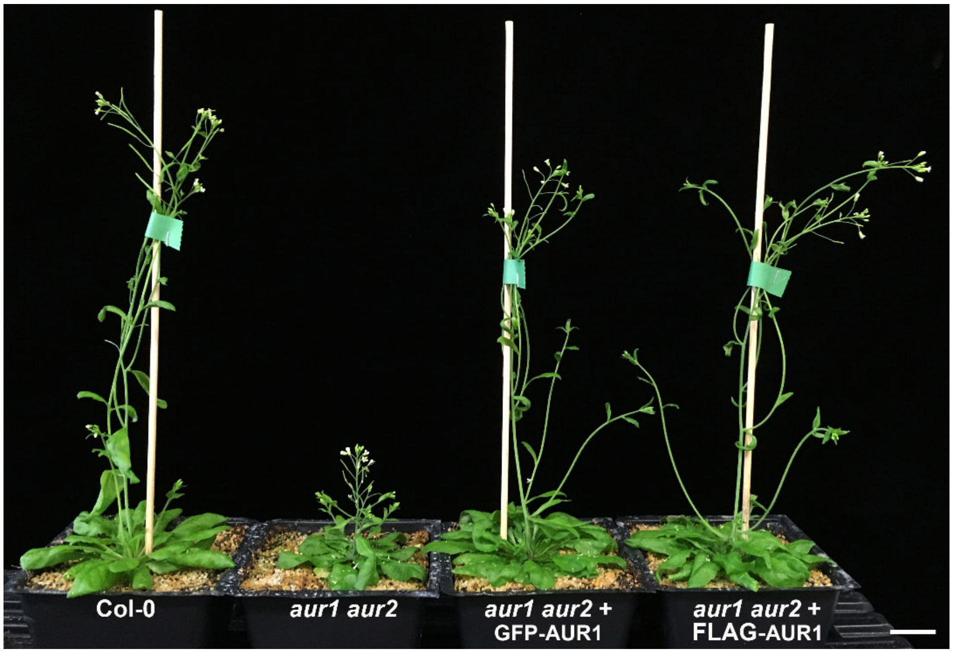

Supplemental Figure S4. Rescuing the *aur1 aur2* double mutant by *AUR1* expression. Compared to the wild-type control, the *aur1 aur2* double mutant exhibits a retarded growth phenotype and produces a dwarf plant. This phenotype can be significantly suppressed when AUR1 is expressed in either a GFP or a FLAG fusion protein. Scale bar, 2 cm.

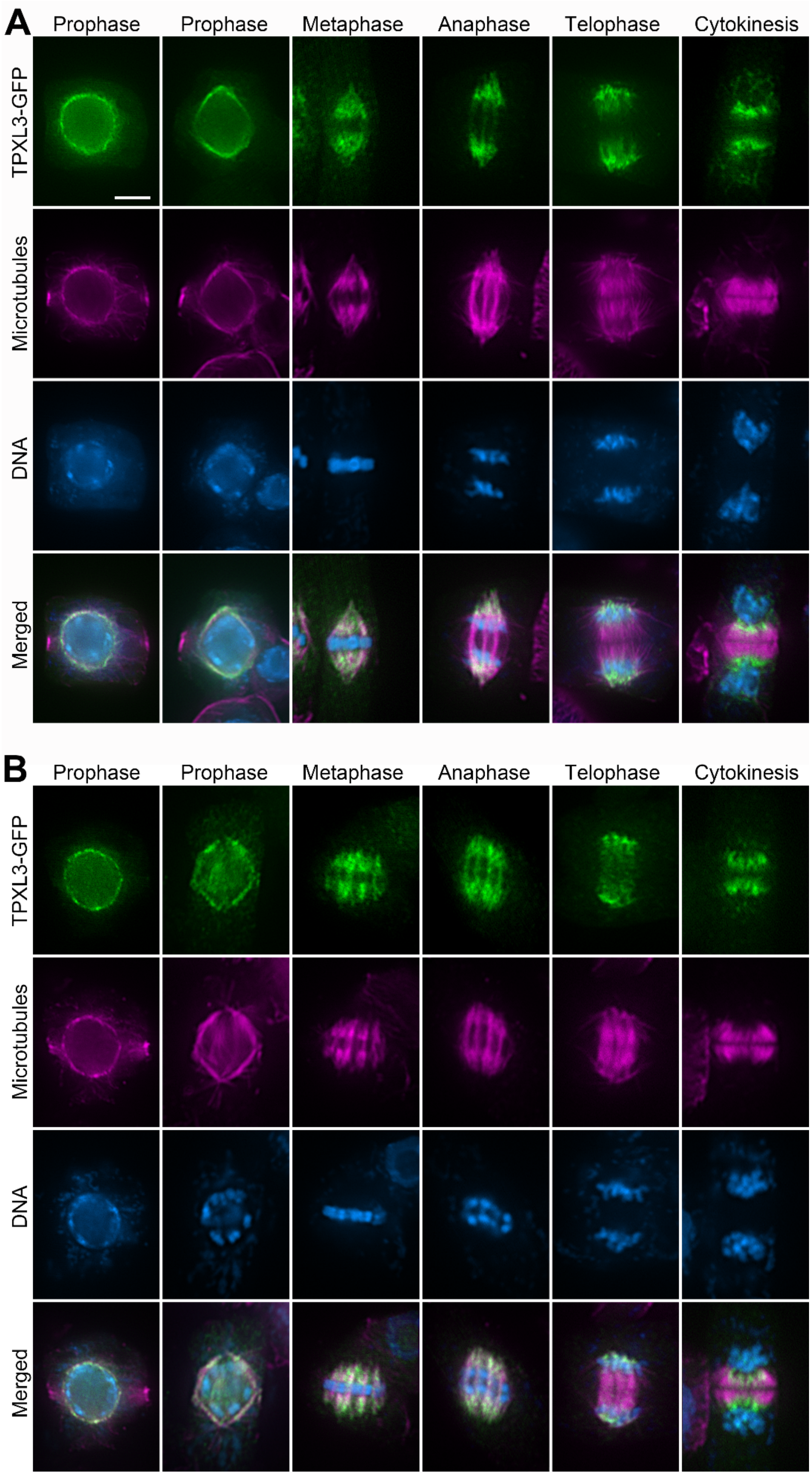
A
Prophase
Prophase
Metaphase
Anaphase
Telophase
Cytokinesis
TPXL3-GFP
Microtubules
DNA
Merged
B
Prophase
Prophase
Metaphase
Anaphase
Telophase
Cytokinesis
TPXL3-GFP
Microtubules
DNA
Merged

Supplemental Figure S5. TPXL3 localization is not altered in the *aur1 aur2* double mutant. (**A**) In the wild-type control cells, TPXL3-GFP exhibits a cell cycle-dependent localization on the nuclear envelope at prophase, as polar caps towards the end of prophase, on kinetochore fibers at metaphase and anaphase, at spindle poles during telophase, and flanking the phragmoplast during cytokinesis. (**B**) in the *aur1 aur2* double mutant, TPXL3-GFP still decorates the prophase nuclear envelope and mitotic microtubule arrays as seen in the control cells that produced spindles of widened poles. Scale bar (applicable to all micrographs), 5 μm.

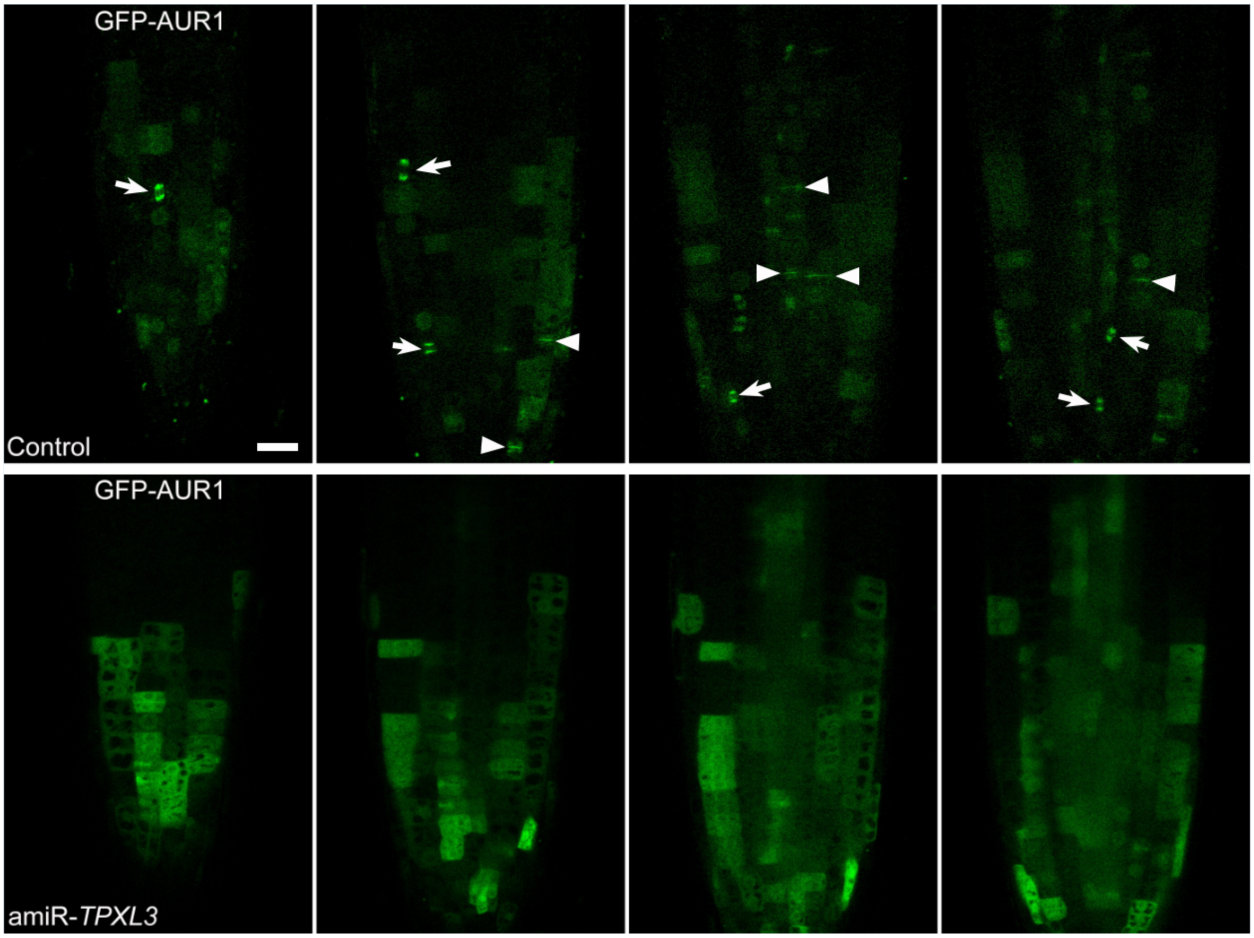

Supplemental Figure S6. Detection of GFP-AUR1 in control and amiR-*TPXL3* root cells. GFP-AUR1 is expressed under the control of the *AUR1* native promoter so that cells with conspicuous fluorescent signals, when compared to those dark ones, are at various stages of mitosis. Both the control and amiR-*TPXL3* mutant roots have four representative optical sections shown here. In the control root, GFP-AUR1 decorated both the spindle pole (arrows) and the phragmoplast midlines (arrowheads). In contrast, the amiR-*TPXL3* mitotic cells have diffuse localization patterns of GFP-AUR1 in the cytoplasm of mitotically active cells. Scale bar (applicable to all micrographs), 20 µm.

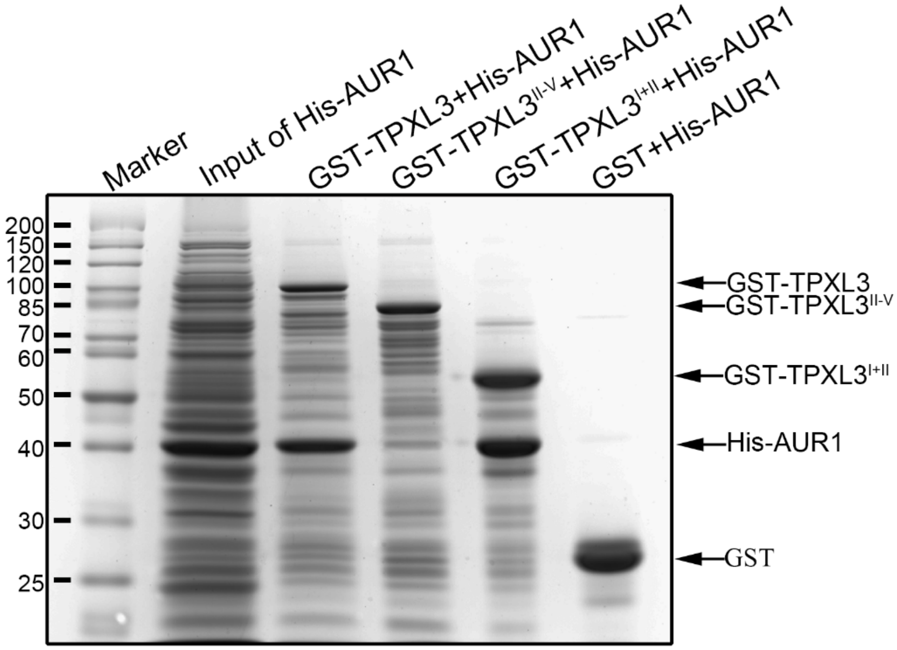

Supplemental Figure S7. Interaction between TPXL3 and AUR1 by the pull-down assay. A His-AUR1 fusion protein from the bacterial extract (input of His-AUR1) can be captured by GST-TPXL3 using an immobilize glutathione column (GST-TPXL3 + His-AUR1). The deletion of domain I abolished the association (GST-TPXL3$^{II\text{-}V}$ + His-AUR1), but the truncated GST-TPXL3^{I+II} is able to do so (GST-TPXL3^{I+II} + His-AUR1). GST alone, as a negative control, is unable to capture AUR1 (GST + His-AUR1). Molecular masses of the standard markers are shown in kDa on the left.

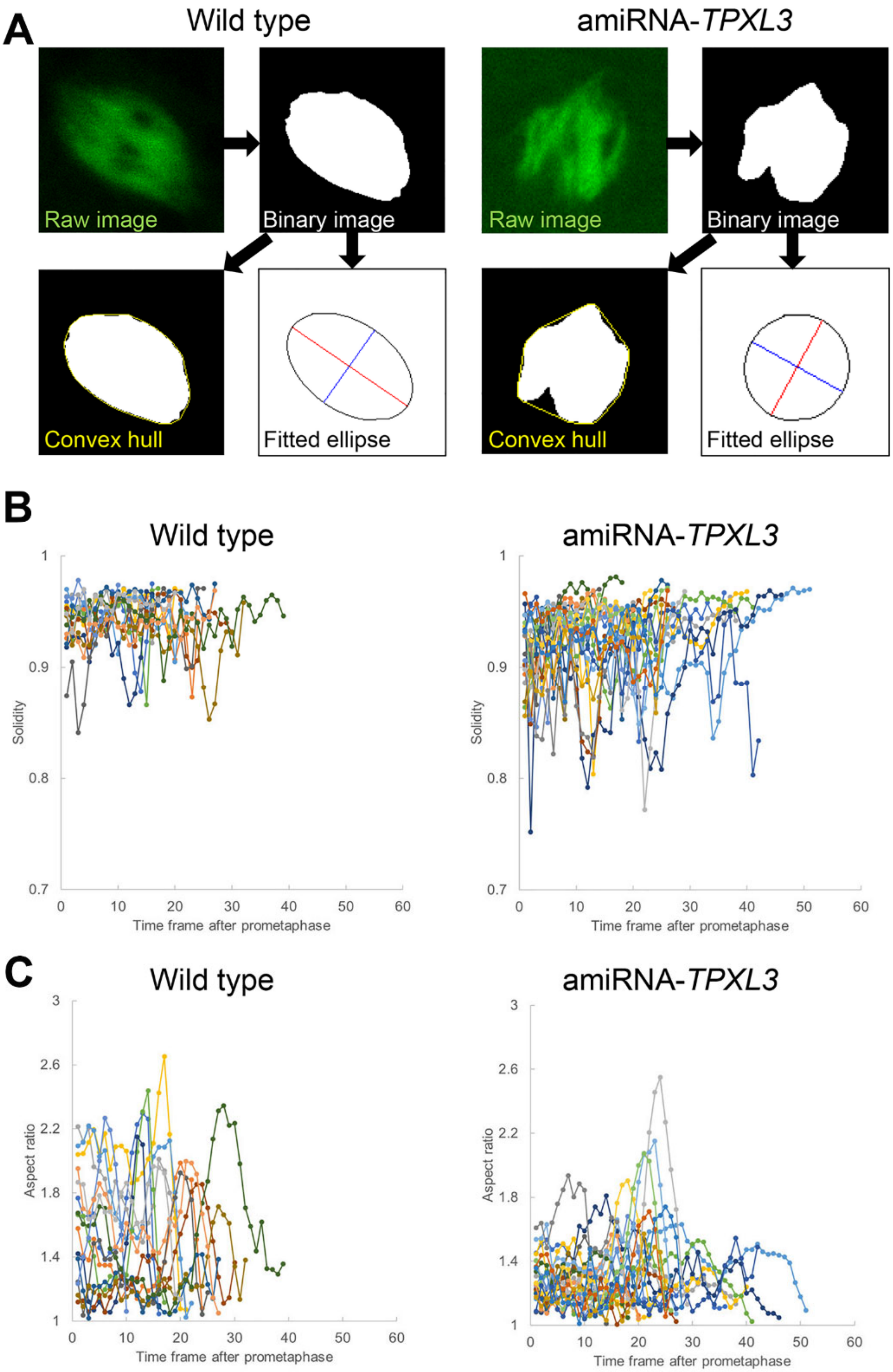

A
Wild type
amiRNA-TPXL3
Raw image
Binary image
Convex hull
Fitted ellipse
B
Solidity
Time frame after prometaphase
C
Aspect ratio

Supplemental Figure S8. Image processing and quantification of the spindle morphology. (**A**) A schematic workflow of image processing to measure solidity and aspect ratio of the spindle. Representative images of wild-type (left) and amiRNA-*TPXL3* cells are shown. Raw confocal images were binarized with the Gaussian filter (Sigma = 2 pixels) and Otsu's thresholding. To measure the solidity, convex hull (yellow closed line) was determined. Solidity is defined as the ratio of the spindle area (white region) to the convex hull area (inside the yellow closed line). To measure the aspect ratio, the binarized spindle regions were fitted to ellipse (black line ellipse). Aspect ratio is defined as the ratio of the pole-to-pole length (red lines) to the width in the equatorial plane (blue lines) of the fitted ellipse. Solidity and aspect ratio are used as the indicators of spindle convergence and elongation, respectively. (**B**) Time-course of the spindle solidity from prometaphase to early telophase in wild type (left; N= 15) and amiRNA-*TPXL3* (right; N = 23). (**C**) Time-course of the spindle aspect ratio from prometaphase to early telophase in wild-type (left; N= 15) and amiRNA-*TPXL3* mutant cells (right; N = 23).

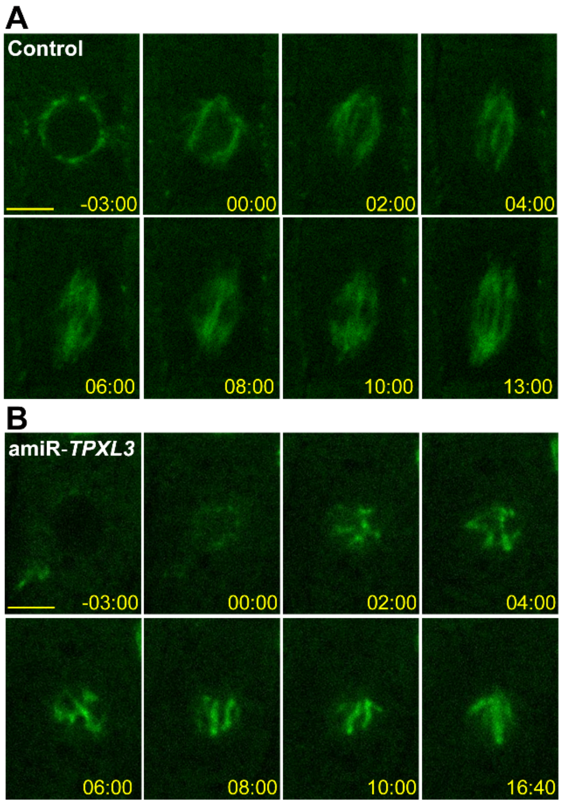

Supplemental Figure S9. The mitotic spindles in the amiR-*TPXL3* cells are hypersensitive to oryzalin. The wild-type (control) and amiR-*TPXL3* cells are imaged in the presence of 100 nM oryzalin. Time stamps are in min:sec, and 00:00 is assigned at nuclear envelope breakdown. Snapshots are taken from Supplemental videos 4 and 5. (**A**) The control cell shows prominent microtubules concentrated on the nuclear envelope (-03:00). Following nuclear envelope breakdown, newly formed microtubules are organized into bipolar arrays that are remodeled with one reflecting anaphase onset (08:00). Later anaphase spindle arrays reflect the shortening of kinetochore fibers (10:00 and 13:00). (**B**) In the amiR-*TPXL3* cell after the oryzalin treatment, microtubule bundles are formed after nuclear envelope breakdown. But these randomly positioned microtubule bundles remain in disorganized patterns even at 16:40 after nuclear envelope breakdown. Scale bars, 5 μm.

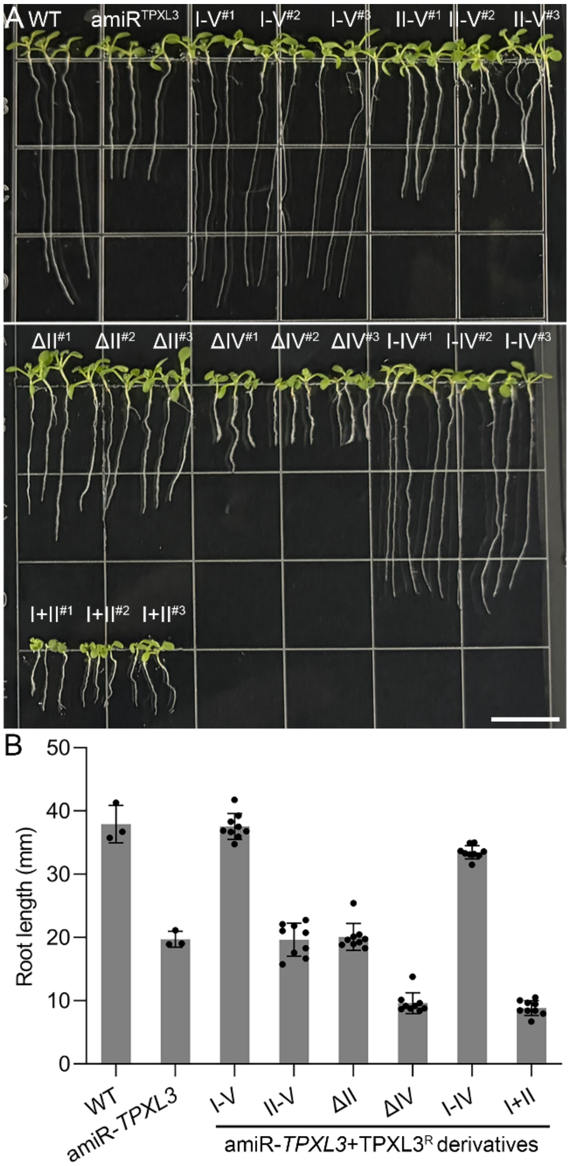

Supplemental Figure S10. Quantitative assessment of root growth phenotypes associated with the expression of the TPXL3 derivatives. (**A**) Representative images show 10-day-old seedlings derived from three independent transgenic events for each construct depicted in Fig. 7. (**B**) Quantitative analysis of plant root length (in mm) illustrates the impact of expressing the TPXL3 derivatives described in Fig. 7. Data are collected from seedlings derived from the three independent transgenic events as shown in (A). Sample sizes are: n=3 for WT and amiR-*TPXL3* seedlings, and n=9 for all other genotypes. The values of mean ± S.D. are shown. Scale bar (applicable to both images), 1 cm.

Supplemental Table S1. Primers used in this study

Gene	Primer Name	Purpose	Oligonucleotide sequence (5' to 3')
At4g22860	816624LP	LP for SAIL_350_B08	ATCAGTGATTTTCTTCGGTTAGTTG
	816624RP	RP for SAIL_350_B08	TATTTTGTTTTGCTAATTGGCTAGC
	480B12LP	LP for GABI_480B12	GTTCAGAGGACGAGCTTTAAAAAC
	480B12RP	RP for GABI_480B12	TTCTCTGTTTCAGGTTGCTGATAC
	22860F	F primer to amplify genomic TPXL3 sequence for cloning into the pENTR/D-TOPO vector	CACCCCTTGTCTTCTTACACAGTAC
	22860R	R primer for amplifying genomic TPXL3 sequence for cloning into the pENTR/D-TOPO vector	TGCACCAGCTCCGCGAATGCCTAAGTTCCTGCAAAAT
	ami-TPXL3F	F primer for TPXL3 artificial miRNA counstruction	TGTATTTCGGAGTGCTCTTTTTCCCATGATGATCACATTCGTTATCTATTTTTTGGGAAAAAGATCACTCCGAAA
	ami-TPXL3R	R primer for TPXL3 artificial miRNA counstruction	AATGTTTCGGAGTGATCTTTTTCCCAAAAAATAGATAACGAATGTGATCATCATGGGAAAAAGAGCACTCCGAAA
	rami-TPXL3F	F primer for counstruction of TPXL3-GFP vector which resistant to artificial miRNA	CGtAAgAAatcCACaCCaAAgCTACCAGAATTCCAAgtaagcct
	rami-TPXL3R	R primer for counstruction of TPXL3-GFP vector which resistant to artificial miRNA	cTTtGGtGTGgatTTcTTaCGGATGGGCAATGAAGGAGCATCA
	TPXL3F	F primer to amplify TPXL3 CDS for cloning into the pENTR/D-TOPO vector	CACCATGGAGATGGATGAAGATATGG
	TPXL3R	R primer to amplify TPXL3 CDS for cloning into the pENTR/D-TOPO vector	AGCTCCTGCACCGCGAATGCCTAAGTTCCTCCTA
	$TPXL3^{1-415}F$	F primer for construction of pENTR-$TPXL3^{I-IV}$	CGGGATCCGGTGCAGGAGCTAAGGGTGGG
	$TPXL3^{1-415}R$	R primer for construction of pENTR-$TPXL3^{I-IV}$	CGGGATCCAAAACTAAACTCCAAAGGTAC
	$TPXL3^{62-189}F$	F primer for construction of pENTR-$TPXL3^{II}$	CACCATGACAAAACTGTTACTAAGAGAG
	$TPXL3^{62-189}R$	R primer for construction of pENTR-$TPXL3^{II}$	TGCACCAGCTCCATATGGAATTTACTGGCAT
	$TPXL3^{190-415}F$	F primer for construction of pENTR-$TPXL3^{III+IV}$	CACCATGCAAGTGGATCAAAGTCATG
	$TPXL3^{190-415}R$	R primer for construction of pENTR-$TPXL3^{III+IV}$	TGCACCAGCTCAAAACTAAACTCCAAAGGTA

	TPXL3$^{252-509}$F	F primer for construction of pENTR-TPXL3^{IV+V}	CACCATGACAAAGATTACTATCCCACAG
	TPXL3$^{62-509}$F	F primer for construction of pENTR-TPXL3^{II-V}	CGGGATCCACAAAACTGTTACTAAGAGAG
	TPXL3$^{62-509}$R	R primer for construction of pENTR-TPXL3^{II-V} or pENTR-TPXL3^{IV+V}	CGGGATCCGGTGAAGGGGGCGGCCGCGGAG
	TPXL3$^{\Delta 62-189}$F	F primer for construction of pENTR-TPXL3$^{\Delta II}$	CGGGATCCCAAGTGGATCAAAGTCATGAT
	TPXL3$^{\Delta 62-189}$R	R primer for construction of pENTR-TPXL3$^{\Delta II}$	CGGGATCCTACAAAAGGAGAAGGAGCGTA
	TPXL3$^{\Delta 252-415}$F	F primer for construction of pENTR-TPXL3$^{\Delta IV}$	CGGGATCCCATTCGGAAAAGAGAGTTCAA
	TPXL3$^{\Delta 252-415}$R	R primer for construction of pENTR-TPXL3$^{\Delta IV}$	CGGGATCCCCTGGCATGTTGAGCGTTTT
	GST-TPXL3F	F primer to amplify TPXL3 CDS for Gibson into the pGEX-KG	atccccgggaatttccggtggtggtggtggaATGGAGATGGATGAAGATATGGAAAT
	GST-TPXL3R	R primer to amplify TPXL3 CDS for Gibson into the pGEX-KG	acgatgaataagcttgagctcgagtcgaccTCAGCGAATGCCTAAGTTCCTCCTAG
	GST-TPXL3$^{1-189}$F	F primer to amplify TPXL3^{I+II} for Gibson into the pGEX-KG	AGTAAATTCCATATGTGAggtcgactcgagctcaagcta
	GST-TPXL3$^{1-189}$R	R primer to amplify TPXL3^{I+II} for Gibson into the pGEX-KG	ctcgagtcgaccTCACATATGGAATTTACTGGCATTATTT
	GST-TPXL3$^{62-509}$F	F primer to amplify TPXL3^{II-V} for Gibson into the pGEX-KG	gtggtggtggaATGACAAAACTGTTACTAAGAGAGGAGG
	GST-TPXL3$^{62-509}$R	R primer to amplify TPXL3^{II-V} for Gibson into the pGEX-KG	TAGTAACAGTTTTGTcattccaccaccaccaccggaaatt
	TPXL3qPF	F primer for qPCR analysis of TPXL3 expression	GAAAGTAGGAGACCAAGCGGT
	TPXL3qPR	R primer for qPCR analysis of TPXL3 expression	AATGCCCATGTCTCCTCTGC
At4g11990	079098LP	LP for SALK_079098	TTTTGCGCTTATTTCTCTAATC
	079098RP	RP for SALK_079098	CTGACATACCAAGGGAGCTGTA
	11990F	F primer to amplify genomic TPXL2 sequence for cloning into the pENTR/D-TOPO vector	CACCTAACATAAGTATATATGCAAACAAC
	11990R	R primer to amplify genomic TPXL2 sequence for cloning into the pENTR/D-TOPO vector	TGCACCAGCTCCATTTAGTTGCACCGTTGCTTCGAG

At4g32830	32830F	F primer to amplify genomic AUR1 sequence for cloning into the pENTR/D-TOPO vector	CACCTTCTCTGTGGACTCCGTATCAT
	32830R	R primer to amplify genomic AUR1 sequence for cloning into the pENTR/D-TOPO vector	AGCTCCTGCACCAACTCTGTAGATTCCAGAAGGAT
	L32830F	F primer for linearizing pENTR-genoAUR1	GGTGCAGGAGCTATGGCGATCCCTACGGAGACACA
	L32830R	R primer for linearizing pENTR-genoAUR1	GGAAGAAGAGATCAACCCAACA
	GFP32830F	F primer to amplify eGFP for Gibson into linearized pENTR-genoAUR1	TCTTTCTTTGTTGGGTTGATCTCTTCTTCCATGGTGAGCAAGGGCGAGGAGC
	GFP32830R	R primer to amplify eGFP for Gibson into linearized pENTR-genoAUR1	CTCCGTAGGGATCGCCATAGCTCCTGCACCCTTGTACAGCTCGTCCATGCC
	FLAG32830F	F primer for construction of the Flag-AUR1 vector	ACAAGGATGACGATGACAAGGGTGCAGGAGCTGCGATCCCTACGGAGACACAACA
	FLAG32830R	R primer for construction of the Flag-AUR1 vector	CCTGCACCCTTGTCATCGTCATCCTTGTAGTCCATGGAAGAAGAGATCAACCCAA
	GST-AUR1F	F primer to amplify AUR1 CDS for Gibson into the pGEX-KG	atccccgggaatttccggtggtggtggtggaATGGCGATCCCTACGGAGACACA
	GST-AUR1R	R primer to amplify AUR1 CDS for Gibson into the pGEX-KG	acgatgaataagcttgagctcgagtcgaccTTAAACTCTGTAGATTCCAGAAGGATCAG
	His-AUR1F-*BamHI*	F primer to amplify AUR1 CDS for cloning into the pET28a	CGGGATCCATGGCGATCCCTACGGAGACACAA
	His-AUR1R-*SalI*	R primer to amplify AUR1 CDS for cloning into the pET28a	GCGTCGACAACTCTGTAGATTCCAGAAGGATC
	AUR1F	F primer for amplifying AUR1 CDS	ATAAGAATGCGGCCGCCCCCTTCACCATGGCGATCCCTACGGAGACAC
	AUR1R	R primer for amplifying AUR1 CDS	AGGCGCGCCCACCCTTAACTCTGTAGATTCCAGAAGGATC
